# The SHOW RESPECT adaptable framework of considerations for planning how to share trial results with participants, based on qualitative findings from trial participants and site staff

**DOI:** 10.1186/s13063-024-08291-7

**Published:** 2024-07-10

**Authors:** Annabelle South, Claire Snowdon, Eva Burnett, Barbara E. Bierer, Katie Gillies, Talia Isaacs, Matthew R. Sydes

**Affiliations:** 1 MRC CTU at UCL, Institute of Clinical Trials and Methodology, UCL, London, UK; 2 London School of Hygiene and Tropical Medicine, London, UK; 3 Patient Representative On Studies at the MRC CTU at UCL, Institute of Clinical Trials and Methodology, UCL, London, UK; 4 Harvard Medical School and Brigham and Women’s Hospital, Boston, MA 02115 USA; 5 Health Services Research Unit, University of Aberdeen, Aberdeen, UK; 6 UCL Institute of Education, UCL, London, UK

**Keywords:** Feedback of results, Communicating results, Researcher perspective, Researcher–participant relations, Trial conduct, Trial ethics

## Abstract

**Background:**

Sharing trial results with participants is a moral imperative, but too often does not happen in appropriate ways.

**Methods:**

We carried out semi-structured interviews with patients (*n* = 13) and site staff (*n* = 11), and surveyed 180 patients and 68 site staff who were part of the Show RESPECT study, which tested approaches to sharing results with participants in the context of the ICON8 ovarian cancer trial (ISRCTN10356387). Qualitative and free-text data were analysed thematically, and findings used to develop the SHOW RESPECT adaptable framework of considerations for planning how to share trial results with participants. This paper presents the framework, with illustrations drawn from the Show RESPECT study.

**Results:**

Our adaptable ‘SHOW RESPECT’ framework covers (1) Supporting and preparing trial participants to receive results, (2) HOw will the results reach participants?, (3) Who are the trial participants?, (4) REsults—what do they show?, (5) Special considerations, (6) Provider—who will share results with participants?, (7) Expertise and resources, (8) Communication tools and (9) Timing of sharing results. While the data upon which the framework is based come from a single trial, many of our findings are corroborated by findings from other studies in this area, supporting the transferability of our framework to trials beyond the UK ovarian cancer setting in which our work took place.

**Conclusions:**

This adaptable ‘SHOW RESPECT’ framework can guide researchers as they plan how to share aggregate trial results with participants. While our data are drawn from a single trial context, the findings from Show RESPECT illustrate how approaches to communication in a specific trial can influence patient and staff experiences of feedback of trial results. The framework generated from these findings can be adapted to fit different trial contexts and used by other researchers to plan the sharing of results with their own participants.

**Trial registration:**

ISRCTN96189403. Registered on February 26, 2019. Show RESPECT was supported by the Medical Research Council (MC_UU_12023/24 and MC_UU_00004/08) and the NIHR CRN.

**Supplementary Information:**

The online version contains supplementary material available at 10.1186/s13063-024-08291-7.

## Background

Late-phase randomised controlled trials often require hundreds or thousands of participants to detect meaningful differences in outcomes. In order to successfully answer their research questions, trialists must recruit volunteers to take part, often asking participants to accept risk and/or inconvenience, with the aim of improving treatment, care or prevention of disease for future patients.

Sharing results with trial participants is an ethical imperative [1] and is recommended by authorities that govern the conduct of clinical trials. The World Medical Association’s Declaration of Helsinki, which outlines the principles for ethical conduct of medical research involving human participants, states that ‘*all medical research subjects should be given the option of being informed about the general outcome and results of the study*’ [2]. In the UK, the Health Research Authority (HRA) recently published guidance saying that participants have the right to know the findings of research in which they have taken part, and that sharing results directly with participants can help ‘*build trust, show respect and helps participants feel valued*’ [3].

There is evidence from a broad array of studies that most participants want to be offered the opportunity to receive trial results, ranging from 88 to 98% in studies conducted across a range of diseases (cancers, idiopathic scoliosis, internal derangement of the knee, HIV) and geographical settings (including USA, UK, Canada and Uganda) [4–8].

Despite the moral obligation and clear demand from most participants to receive results, in practice, sharing results often does not happen, or is not done well. The UK HRA research transparency report in 2021 states that ‘*90% of clinical trials have not told participants about findings*’ [9]. A survey conducted in 2016 of authors of clinical trial results papers published in 2014–2015 found that only 27% of respondents reported disseminating results to participants, with only a further 13% planning to do so [10]. Even when it does happen, it may not be done in a way that participants can understand. The survey found that 40% of authors who had shared results with participants had shared academic publications, which are not written in a way that is easy for participants to understand [10]. Previous studies have reported that many participants struggle to understand trial results which are shared with them. For example, a study within the context of a breast cancer trial found that only 56% of participants said the results letter was easy to understand [11], and a survey of cancer trial participants found fewer than half reported fully understanding the results [7].

Sharing results with participants is a complex issue, with trialists facing considerable challenges including practical [10, 12–17] and resource barriers [10, 12–15, 18, 19] and concerns about the emotional impact of sharing results [4, 7, 11, 12, 14, 19–22]. It is important that it is done well, as there is potential for harm [13, 23–25]. Little work has been done to compare approaches to sharing results with participants. Show RESPECT assessed approaches to sharing results in a cluster randomised factorial trial, comparing an enhanced versus basic webpage; mailed printed summary versus no printed summary; and email list invitation versus no email list invitation, within the context of an ovarian cancer trial (ICON8) [26]. A major finding was that the mailed printed summary significantly improved patient satisfaction with how results were shared compared to a webpage with or without an email list invitation, without the printed summary [26]. It also showed that these approaches were feasible for site staff to implement [27]. However, Show RESPECT was carried out within only a single trial, in a particular clinical and geographical setting, with a particular set of results to communicate, so the generalisability of these results is unclear. It is likely that there is no one-size-fits-all approach to sharing results, so it is important that trial teams draw on guidance that offers a sound structure that can be adapted to fit the specific context and requirements of their own trial and trial participants.

One of the aims of the Show RESPECT study was to develop guidance for trialists, based on our results. We realised that findings from Show RESPECT [26, 27] could be used to derive an adaptable framework of factors for trialists to consider when planning how to share results with trial participants. This paper presents the framework, based on and illustrated with qualitative insights from data collected from site staff and patients who took part in the Show RESPECT study.

## Methods

Show RESPECT was a mixed methods study, comprised of a factorial cluster randomised controlled trial within a trial to assess multiple approaches to communicating trial results, and an embedded explanatory qualitative study. The full protocol for the study is available online [28]. Results from Show RESPECT with regard to participant satisfaction with how the results were shared, and the resources required from sites and the clinical trials unit (CTU) to implement these approaches, have been reported previously [26, 27] and the qualitative results reported in this paper have been published as part of a doctoral thesis [29]. Show RESPECT took place within the ICON8 ovarian cancer chemotherapy trial (ISRCTN10356387) [30]. As the methods have been reported previously, we do not duplicate this information in this article, but have included it as Additional File 1. This paper reports qualitative results from data collected from trial participants and site staff who had been involved in sharing or receiving results. The Standards for Reporting Qualitative Research checklist for this paper can be found in Additional File 2.

Information about patient and public involvement in this study and the context of the ICON8 trial is available in Additional File 1.

### Qualitative data collection

The main source of qualitative data for Show RESPECT was semi-structured interviews with ICON8 patients and site staff who had been involved in sharing the ICON8 results with patients. In addition to the qualitative interviews, qualitative data were collected by free-text questions on the questionnaires that were completed by patients (after receiving results) and site staff (immediately after sharing results and 2–3 months later). The topic guide, questionnaires, details of how these were administered, and researcher characteristics and reflexivity can be found in our previous publications [26, 27, 29, 31]. Further information about our qualitative data collection is available in Additional File 1.

### Sampling and participants

We used a purposive sampling approach for the semi-structured interviews with both participants and site staff, allowing us to collect data from respondents with a range of characteristics that may be related to their experiences and views on sharing results. For ICON8 patients, this included age, education level, frequency of internet use and reported satisfaction with how the ICON8 results were shared, while for site staff this included their role and number of ICON8 patients at the hospital at which they work. For both groups, we included which interventions their hospital had been randomised to. Further information about our sampling approach and participants is available in Additional File 1.

### Qualitative analysis

We used a reflexive thematic analysis approach [32], with a critical realist stance (taking the ontological position that an external reality exists that is independent of our beliefs and understanding, but that our knowledge of that external reality is influenced by our historical, social and cultural situation), to analyse the data. The findings reported in this paper are further findings from the analysis carried out for our previous papers [26, 27], rather than a separate re-analysis of the Show RESPECT data. Further details of our analysis methods are available in Additional File 1.

### Developing the framework

We shared our findings around what influenced the experience of patients and site staff around receiving/sharing trial results at a patient and public involvement meeting with women who were taking part in ovarian cancer treatment trials. We held meetings with site staff who had been involved in sharing the ICON8 results with participants, and met with the ICON8 trial team. We also held seminars at three clinical trials units and presented our findings at a clinical trials conference. At these meetings, we discussed with these key stakeholders the implications our results, how they might be transferred to trials in other settings, and recommendations they would make for future trials. Based on the themes, sub-themes and high-level codes from our data, and the stakeholder discussions, we developed a long list of considerations that we believe trial teams should take into account when planning how to share results with trial participants, either because it came from our qualitative data, or was raised as an additional consideration during our discussions with stakeholders. We grouped related considerations together into categories and organised the categories so the initials spell a memorable phrase (SHOW RESPECT).

To explore how useful the framework was for a trial that was very different to the ICON8 trial in which we carried out Show RESPECT, we applied it to CHAPAS-4 (ISRCTN 22964075) [33]. This was done by a single researcher (AS) who worked with the study teams and (for CHAPAS-4) community representatives to consider the factors identified in the framework, and how they affect the communication of results to participants for these trials. AS had been involved in CHAPAS-4 from the proposal development stage, so was familiar with the study.

## Results

A description of the patient and site staff participants in Show RESPECT is reported in our previous papers [26, 27]. A table showing a summary of their characteristics can be found as Additional File 3.

The adaptable framework of factors trialists should consider when planning how to share results with trial participants is shown in Table 1, with illustrative quotes from the Show RESPECT data. An editable template with the adaptable framework can be found online [34]. The framework covers supporting and preparing participants to receive results; how the communications tools will reach participants; who the trial participants are; what the results show; special considerations; who will provide results to participants; the expertise and resources the trial team have access to for sharing results; which communication tools will be used; and timing of results communication. Additional File 4 shows how the framework items relate to the qualitative themes, sub-themes and high-level codes from Show RESPECT. Additional File 5 illustrates these factors with findings from the qualitative interviews conducted during Show RESPECT.

**Table 1:** Factors to consider when planning how to share results with trial participants, with illustrative quotes from the Show RESPECT study

	**Considerations**	**Additional prompts**	**Illustrative quotes**
S	**S**upporting and preparing trial participants to receive results	How will you prepare participants for receiving results? • What did your Patient Information Sheet say about how and when results will be shared? • How will you inform participants that the results are available? Will you use an opt-in or opt-out approach? • Are most of your participants likely to want to know the results (if so, an opt-out approach may be best)? • How and when will you give participants the choice of whether to receive results? How will you provide support to patients who have additional questions or concerns about the results? • Are participants still in follow-up? Can they still access support from their research nurse/doctor? • What other support is available to them to help understand and process the results?	‘*I think it would be a really good idea going forward to, you know, ask patients if they want the results when consenting to the clinical trial, And, again, ask them once they’ve completed the treatment if they’d still like to receive the results.*’ HLRNI03: research nurse, large site ‘*I think they just wouldn’t care enough to call to opt in, whereas if they really didn’t want the results then I think they’d be more willing to call or take the time out of their day to do that.*’ CLTCI04: trial coordinator, large site ‘*That was the thing, I think, we were slightly concerned about was, well, what if that raises questions, which again is why we put that you know, compliments slip in… you know, do phone us if you’ve got any issues with it or queries or anything.*’ DMRNI02: research nurse, medium site ‘*I suppose to some extent, it’s on the research nurses because the two that I saw are really great. I get on really well with them and I don’t feel afraid to ask them any questions. But I think that’s more a personal thing really. It’s quite difficult for somebody if they can’t relate to the people they’re seeing, for whatever reason, obviously the next best option would be to have some paper to take away and read in their own time.*’ DMI01: patient, medium site, close relationship to site staff ‘*Particularly if sharing the results of the study reawakens emotions that were present initially, at initial diagnosis or initial treatment. Having some ability for that patient to get some additional emotional and psychosocial support is important. But also, if they have questions about the results and for whatever reason they are unable to get appropriate answers from their investigators, being able to go onto patient forums or nurse advisor lines, I think will be important for a proportion of trial participants.*’ HLCLI02: oncologist, large site
HO	**HO**w will the communication tool(s) reach participants?	Which communication mediums are likely to be accessible to your participants? How can you make sure the results are accessible to all your participants? Where will participants receive results? • Will participants prefer to receive results at the clinic, where support may be immediately available, or in the privacy of their own homes, where they can process it in their own time?	‘*I know quite a few that wouldn’t bother and don’t like things online anyway… I hate to say it but even my age group don’t like getting things on email. They like it in their hand.*’ BMI02: patient, medium site, aged 71 or older ‘*I think if the results had been bad (in terms of I would have had to tell them that this treatment arm is better than your treatment arm), I think how they react to that, the clinic isn’t as private as you would want. If I had to tell them to their face I don’t think it would have been as good as me just sending them something on the web page and then putting at the bottom, you know, they needed any further support or whatever they can just call me. Instead of having them be in front of everyone reacting to it, be able to read it on their own time in their own space and react how they would want to react.*’ CLTCI04: trial coordinator, large site
W	**W**ho are the trial participants?	What are the demographic characteristics of your trial participants? • What is their: age, socio-economic status, education level, health literacy, computer literacy, access to the internet? How well are your participants likely to be? • How is their health at the time of receiving results? • How was their health and experience of adverse events or side-effects during the trial? What expectations do your participants have around receiving trial results? • What did you put in your Patient Information Sheet about whether/when results would be available? • Do you need to get ethics approval for any changes to how you plan to share results? What will participants want to do with the results? • Will participants want to keep results for future reference? • Will participants want to share results with others?	‘*You’ve got to look at the age group of the patient. So everybody is individual, so like, if you are looking at maybe 65 and above, they would mostly prefer written summaries. Whereas the younger group will want the website.*’ BMRNI04: research nurse, medium site ‘*If there really was a finding that actually, people were living longer and I’d got secondaries or something, yes, I would have liked to have been spoken to about that rather than finding out on the website.*’ CLI01: patient, large site ‘*I think you’d probably have to be a bit more careful in terms of sharing results with patients who were very unwell and closer to their end of life. Particularly if they’ve reached the point where they are, I suppose, have come to terms with the terminal nature of their illness. Sharing information that might bring back difficult memories at that point, might be more difficult. I think it’s probably still best practice that if we do have that information available and we’re seeing the patient, that we ask them whether they want to know about the outcome of the trial.*’ HLCLI02: oncologist, large site ‘*I assumed that I would never know the results, that it would be… Well first of all I thought well I’ll probably be dead anyway, but no, I didn’t think they would be available. I thought trials probably went on for much longer, and that they would wait until people died before they assessed it.*’ GSI01: patient, small site ‘*It’s something that I think is becoming more important. A lot of our patients are becoming more empowered. They’re wanting to seek more information. Treatment of cancer is becoming more complex, often patients will survive for longer and live with their cancer as a chronic illness. Probably there are more trial participants who are keen and interested in finding out the results of studies that they have taken part in, in the past. It is becoming a greater priority for us to engage with them in this setting.*’ HLCLI02: oncologist, large site ‘*It was easy to read [the printed summary] over a period of time and I could keep a copy without finding a printer.*’ BMQ05: patient, medium site
RE	**RE**sults—what do they show?	What do your trial results show? • Will it be seen as good/bad/neutral news by some/all participants? How complex are your results? • Are your trial results complex (e.g. there is important heterogeneity between sub-groups, or do different outcomes go in different directions)? Will the results have implications for the participants’ or their families’ future health or care?	‘*I think, when there’s some really good results a doctor always feels that’s what they want to tell their patients. Whereas if there’s a marginal benefit, then you’re likely to not really want to say too much of the results.*’ EBLMCLI02: oncologist, large and medium sites ‘*If it went into my head that I was going to see more bad news about my participating group I might be less inclined to want to see a written report and just a referral to a website. Because this is almost… When you receive this [printed summary] you have to look at it whereas with the website you may think, oh, I’m not going to bother. You can ignore it more easily if you feel that your group is not going to have any more good news or better results.*’ BLI01: patient, large site ‘*It would depend if it raised more questions perhaps. So maybe a clinician would have been better suited I suppose, if it was going to have that effect. Maybe the clinician giving a paper and discussing it in clinic maybe better for them than obviously reading it at home on their own.*’ GSTCI03: trial coordinator, small site
S	**S**pecial considerations	Have things happened over the course of the trial that need to be taken into account? For example: • Were there changes to the trial over the course of the trial that need to be explained? • Are there results from other trials that need to be taken into account when communicating the results of this trial? • Has the trial closed early for efficacy, for safety or for accrual issues? • Has the trial received negative publicity?	‘*I was [surprised] actually because I thought that the reason this was being done was because the Japanese women had found it easier and had found it better to have a more gentle approach. And I saw from this that it wasn’t only the UK, there were other hospitals throughout the world, so it might be something to do with the Japanese diet or their way of life, their whatever, it could be a lot of environmental factors. So, yes, it did surprise me.*’ CLI01: patient, large site
P	**P**rovider—who will provide the results to participants?	How close are relationships between site staff and participants likely to be? • How long were participants in the trial for? • Was follow-up done face-to-face? If face-to-face, was it in person or virtual? • Which organisation or individual was their main point of contact for the trial? • Are the staff members who were their main point of contact still working on the trial? • Does the communication need to be personalised to respect the relationship between site staff and participants? How many participants do sites have? • Will sites with large numbers of participants have sufficient resources to share results individually? Or must other communication approaches be considered?	‘*I think they should do them face to face really. I don’t know if that’s… I mean, the thing is, you build up a relationship with your trial nurses because we see them quite regularly or every time we go for a hospital appointment. So, I think it would be really nice if they presented that themselves, obviously backed up with information. I think because you’re feeling vulnerable anyway and I think if you’ve already built up a relationship with people, then it’s easier to talk to them.*’ DMI01: patient, medium site, close relationship with site staff ‘*The only problem with our site is we recruit so many patients onto our trials, it would be extremely time consuming. Now whether we could, you know, whether the Sponsors could send something out directly to the patients themselves, you know, with an option to receive the results or not. That would probably be a better option, you know, I don’t know. Again, it’s just the volume of patients that we’ve got on trial here. So, it’s a, you know, it’s a lot of information to send out to people, you know, when we’ve got so many patients on trial.*’ HLRNI03: research nurse, large site ‘*I don’t know, because we are a smaller centre and our numbers don’t tend to be like a big teaching hospital, we have that more personal approach, so we know our patients very well, we know the families very well, so it makes it easier for us in that respect. I’m not saying, if it was a teaching hospital you could follow the same principles, but here, we generally have that closeness.*’ CSRNI01: research nurse, small site
E	**E**xpertise and resources—what expertise and resources do you have access to for sharing results?	What budget do you have for sharing results with participants? • Have you budgeted for costs such as printing and postage, filmmaking and web development? What expertise around developing patient-facing communications tools do you have? • Do you have access to expertise on this within the team, through partners or paying for specialist support? Is this activity seen as a priority for CTU and/or sponsor staff? • Is sharing results with participants incorporated in CTU Standard Operating Procedures and trial protocols? (If not, can it be?) Has sharing results been included in agreements with sites? • Do sites know this is a trial activity they are expected to do (if you are planning for the results to be shared by site staff)?	AMRNI05: ‘*If it’s a public post then it can be a bit of an issue for us if postage isn’t supplied because budgets are tight.*’ IV: Is there reluctance in your hospital to cover that sort of cost? AMRNI05: ‘*Yes. Budgets are very tight.*’ (Research nurse, medium site) ‘*Although the majority of sites would actively engage, there may be some sites who feel that it’s an optional extra. And they don’t have the staffing, and the information then doesn’t get out to the participants who would actually like to have that information.*’ HLCLI02: oncologist, large site
C	**C**ommunication tools—which ones will you use?	What will participants want to know? • Can participants who want different levels of information/detail find out what they want to (providing layered information)? What language will your participants understand? • In what languages was the Patient Information Sheet available? • Do you know how to write in plain language? • How will you get feedback from PPI contributors about your draft results summaries? How will you make your information product accessible, welcoming and easy to follow and use? • Do you have the experience in design, numeracy and imagery? • Do you have the skills to do this in-house? • Do you have good templates to base it on? • Do you have the budget to pay for a designer? Can you give participants a choice of information products? • Is it feasible for you to provide more than one way for participants to access the results? Which communication tools will you use for sharing results?	‘*I like that all of them let you know that if you do need further information, there are plenty of ways of obtaining it. And it’s easy to actually get to the stuff. Sometimes it’s a nightmare for when you go online and you’re trying to find something, you can be half an hour searching your way around trying to get to it. But this is easy to get to everything. Support lines, you can’t ask for more than that, can you really? Loads of different support lines and things you can have another look at.*’ BMI01: patient, medium site ‘*The rest of it to me as a member of the public, I can’t do anything with that information; it’s not useful to me. But I would have been interested in as you say a headline result.*’ BLI01: patient, large site ‘*I think the first, I’m not quite sure of the necessity to put all, I don’t know what it means even, under number 1. Study name, it’s quite professionally written from a lay point of view. And all those numbers and letters, goodness knows what that means. ISRCTN:103… You know, for a lay point of view and even from my point of view, I suppose I’m somewhere in between being lay and not lay, it’s gobbledygook really.*’ DLI01: patient, large site, retired nurse ‘*It’s easy, in little chunks, because if I see messy, great big pieces I don’t really want to be bothered, but if it’s in nice chunks like this, that’s how I like it.*’ BMI01: patient, medium site ‘*I quite like the fact that it is slightly a larger font size, and I know that my husband would find it easier to read something like this than something with a smaller font.*’ CSI01: patient, small site ‘*I really like this [Printed Summary]. I think it’s the layout as well … Visually, you can engage someone that’s got an attention span of a two-year-old like me. You can immediately engage someone because of the way something is laid out; they’re more likely to want to read it anyway.*’ GMTCI02: trial coordinator, medium site ‘*I think it is good to offer variety because different things will suit sort of different people.*’ DMRNI02: research nurse, medium site
T	**T**iming—when should results be communicated?	How urgently do results need to be shared? • Are your results likely to receive media coverage? If so, how can you make sure participants don’t first find out results via the media? • Do your results have implications for the future treatment of your participants? • Are participants still in follow-up? If so, is it feasible to integrate sharing results with routine clinic visits or do they need to reach participants sooner? How certain are you that the results/key messages will not change during the peer review process? • Are you sufficiently confident that your key messages are unlikely to change substantively, to share them with participants prior to publication?	‘*If they were to find out that way *via* press coverage because you haven’t let them know that it’s going to be coming out in the public domain, then that might annoy certain individuals. You probably wouldn’t want to find out, like, put ITV on, the news has come out, and then all of a sudden the paparazzi are there talking about this trial. You’d probably be sitting there thinking, I could’ve done with this information earlier, couldn’t I?… Out of respect for the patient really, you should be telling them as early as possible, I can imagine.*’ GMTCI02: trial coordinator, medium site ‘*As long as you are confident that is what is going to be written in the journal. You are sending this information to people who participated in the study. I think it’s better to let them know as soon as you are confident about whatever has been outlined. Like, waiting for a year, they might be dead.*’ BMRNI04: research nurse, medium site

Template for trial teams is available from https://osf.io/9hwmx/?view_only=1fed4585370e4f0a9d43a79d4940cf97

Consideration needs to be given to supporting and preparing participants to receive trial results. This includes what participants are told when they join the trial, and immediately before receiving the trial results. It also includes how participants will be able to access support around dealing with the emotional aspects of processing the trial results, and finding answers to questions they have about the results and their implications. Patients in ICON8 differed in the extent to which they felt comfortable asking site staff for more information or clarification, and their confidence in searching for health information from other sources, such as online. Some patients were part of local support groups for people with cancer, whereas others felt they received sufficient support from family and friends. Still other patients were neither linked to support groups, nor had family or friends they could talk to about their cancer. In this context, both patients and site staff felt that links to further information and support might be useful for some patients (even if not themselves), particularly those with less access to support with processing the results.

Thought also needs to be given to how the communication tool(s) will reach participants, and the accessibility needs of your patient population. Alongside the question of how the results will reach participants is the question of where. Receiving results in the clinic may make support and clarification more easily available but provides less privacy and time for processing the results than patients receiving them at home.

Participant characteristics may affect the appropriateness of different communication approaches. The people taking part in the ICON8 trial were women with an average age of 67 by the time results were available. Four in ten of them used the internet and email less than daily [26]. In this context, printed summaries were viewed as being easy to access for all participants (including older participants and those who are not confident computer users). Other patient characteristics that may affect results communication include education level and health literacy. Non-written forms of communication (such as videos) may be useful for those who do not like to read. Consideration should also be given to what participants are likely to want to do with the results. Many patient interviewees kept folders with all the information they received about the ICON8 trial, to allow them to refer to it for future reference. Printed summaries of the results facilitated this, while email or webpages required printing. Printed summaries also made it easier to share results with others, such as family and friends.

The nature of the trial and its results also affects how results should be shared. ICON8 found no difference between the different chemotherapy schedules tested. In some ways, this made it easier for some patients to receive the results, as although they were disappointed that the trial did not find an improvement in treatment of ovarian cancer, no one was allocated to an inferior arm. The approaches used to share results in Show RESPECT were felt to be appropriate in this context. If the results had been different, with a clear difference between the arms, some patients and site staff felt that there may have been a need to communicate results to people in the group that had done less well overall in a more personal way. This may be less important in trials for less severe conditions than ovarian cancer, where participants have less riding on the results. Similarly, if the results are complex, they may need personal discussion to help patients to understand. One item in the framework that came from engaging with stakeholders rather than directly from the Show RESPECT data was that of ‘special considerations’ that need to be taken into account, such as if the trial had closed early, or experienced adverse media coverage. In ICON8, some patients wanted explanations on why the ICON8 results differed from those of previous similar trials in different settings. Patient and public involvement in the design of tools and processes is essential.

Communication of results takes place within the context of relationships that have developed over the course of the trial between patients and site staff. Participants in ICON8 have been in follow-up for 5 to 8 years, with regular clinic visits during that time. At sites where participants were seen by the same site staff each time, many developed close relationships, almost friendships. Where this was the case, site staff felt uncomfortable sharing the results without some degree of personalisation, so some added personalised cover notes, or called participants to let them know the study results were about to be disseminated. Communication of trial results should consider the strength of relationships developed between site staff and patients, for example allowing a degree of personalisation of how the results are shared where these relationships are close. Some staff at the largest sites did not know participants so well and felt uncomfortable telephoning patients out of the blue. There may be less need for personalisation in trials with shorter follow-up, or with follow-up that does not involve face-to-face visits with consistent staff over time, or when results come from staff other than those who had developed relationships with participants.

When considering the expertise and resources needed for sharing results with participants, thought needs to be given to the skills, staff time and budget needed for the development of the information product (e.g. writing the content in plain language, patient and public involvement, scientific review to ensure the summary is accurate, and technical skills required for the chosen communications tools); distribution of the results (e.g. site staff time for posting information, costs of distribution [e.g. printing, postage]) and supporting participants and dealing with queries. Our previous report describes the resources required from sites and the clinical trials unit for sharing the results in the ways tested in Show RESPECT [27]. Budget or staff time limitations may rule out certain approaches to sharing results, if they have not been included as part of the initial budget for and funding of the trial.

Choice of communication tools will be influenced by the factors described above. Patient and public involvement is important in helping to make these decisions. In addition to deciding what type of communication tool(s) to use, consideration needs to be given to the content of that tool. It should include the language(s) used and the appropriate reading level for the target audience (if a written tool is used). It should also include consideration of how to make the information attractive and easy to use (which may require input from design specialists). It may be appropriate to offer participants a choice of communication tools, possibly with different levels and forms of information.

The final factor for consideration is timing—when should the results be communicated to participants? This will depend on the point at which the research team are confident that the messages for participants are unlikely to change, and whether (and when) the results are likely to receive media or social media attention, to avoid participants finding out the results from others before hearing from the trial team. It should take into account when results will be released to other audiences (e.g. via conferences, peer-reviewed publications and public trial databases and registries), and associated embargoes and deadlines (such as the European Medicines Authority requirement to post a summary of results within a certain time period from the end of the trial).

Practical examples of the application of the framework to two very different trials can be found in Table 2: the ICON8 ovarian cancer trial in which Show RESPECT was conducted, and the CHAPAS-4 paediatric HIV treatment trial, which was conducted in Uganda, Zambia and Zimbabwe.

**Table 2:** Applying the SHOW RESPECT adaptable framework to the ICON8 and CHAPAS-4 trials

Considerations	ICON8	CHAPAS-4
**S**upporting and preparing trial participants to receive results	The ICON8 Participant Information Sheet told participants that, when results were available, a summary of the results would be available from their doctor and on the MRCCTU website. However, participants will have received that several years ago, so may need reminding that they will receive the results. They will also need information about how the results will be shared, as this was not included in the PIS There is no reason to think that ICON8 participants are less likely to want to know the results than participants in previous studies, so it is probable that most will want to know. An opt-out approach is likely to be suitable for this. A written update for participants (sent by post, as participants are not attending clinic frequently at this stage of the trial) could be used to inform them the results are available, how to access them, and how to opt out. Site teams could keep track of opt-outs, as they will be responsible for sharing results with participants (see ‘Provider’ below) Most participants are still in follow-up for their trial and have access to support from their research nurse or doctor. There is also support available from ovarian cancer charity helplines. Both these sources of support should be highlighted in the results summary. Some research nurses may want to phone participants before or after the results are shared, to check whether they need additional support. However, this might not be feasible for all sites (particularly not for sites with large numbers of participants)	The Patient Information Sheet informed participants that a summary of results would be shared via the CTU website, and the results would be published in a medical journal. They were informed that they would be invited to a meeting at their trial centre, where results would be shared with them Based on previous paediatric HIV trials in these settings, we expect that most participants and their families will be keen to hear the results, and the meetings are likely to be well-attended. Sites will contact the participants’ carers by telephone to invite them to the meeting Providing the results at a participants meeting will enable participants and their caregivers to ask questions and get support if required. While children may no longer be attending the study clinics, they are still attending routine HIV clinic visits, so they can access further support from the counsellors, nurses and doctors at these clinics
**HO**w will the communication tool(s) reach participants?	The participant population for ICON8 is mostly older women, who are less likely than the general population to have regular access to the internet. In this context, relying solely on electronic means of communication would be likely to make the results inaccessible for some participants. However, electronic communication media (webpages and email) may be appropriate for some participants Most participants are not visiting study clinics very frequently, so to avoid unnecessary delays in sharing results we will mail them information about how to access the results	Participants and families in CHAPAS-4 trial may not have access to the internet or email, making these methods not ideal for sharing results. The postal service may not reach all participants. Participants regularly visit clinics, so that is a potential way to reach them, as is inviting them (by telephone) to attend a face-to-face meeting
**W**ho are the trial participants?	Participants in the ICON8 trial were females with an average age of 67 at the time results were available. Education level, computer literacy and access to the internet are likely to vary widely between participants Most participants in ICON8 had stage IIIC or IV disease, meaning the cancer had spread outside the pelvis and, in the case of stage IV, to other organs. In the UK, 5-year survival for stage III disease (which includes less severe disease than IIIC) was 27% and 13% for stage IV disease, for women diagnosed during the time period during which ICON8 recruited participants. The chemotherapy regimens used in the ICON8 trial are associated with significant side-effects The Participant Information Sheet informed participants that a summary of results of the trial would be prepared, available from their doctor and published on the CTU website Many participants in ICON8 kept copies of information they were given about the trial, so would be likely to want to keep the results for future reference. They may also want to be able to share results with family members and friends	Participants in the CHAPAS-4 trial were children living with HIV aged 3–15 years in Uganda, Zambia and Zimbabwe. Their parents or primary caregivers gave consent for them to initially join the trial (some participants have since given consent themselves, when they became old enough). Not all participants or parents/caregivers will be literate. Access to the internet is likely to be limited for many participants, although some participants, parents or caregivers may have smartphones Most children in CHAPAS-4 are in good health, and side-effects were not a major problem for most children in the trial Parents and children were told in the Participant Information Sheet that they would be able to receive the results at a participants meeting Participants and families are unlikely to want to share the results with others due to the stigma around HIV. For similar reasons, they may not wish to keep physical copies of the results
**RE**sults—what do they show?	The ICON8 results showed no benefit from the two experimental regimens. This may be a relief for those in the control arm, who have not missed out on a superior treatment. However, it is likely to be disappointing to participants who were hoping the trial would improve the treatment of ovarian cancer The results were not particularly complex, with no important heterogeneity or conflicting results	The CHAPAS-4 trial found that newer antiretroviral combinations including tenofovir alafenamide and dolutegravir are superior to older second-line options for children living with HIV. In addition, CHAPAS-4 found that combinations including the drugs darunavir or atazanavir are excellent alternative options. Overall, children in the CHAPAS-4 trial did very well. Very few children experienced their disease getting worse, or were hospitalised. The results of each individual comparison are not particularly complex, with no important heterogeneity or conflicting results These results reinforce the current WHO recommendation of dolutegravir-based regimens being preferred second-line regimen for children. They also provide new evidence for use of tenofovir alafenamide in second-line combinations for children
**S**pecial considerations	The trial had not closed early, nor received negative publicity When participants joined the trial, the PIS informed them that a previous study in Japan had found benefit from weekly chemotherapy, so results communication needs to take this into consideration	The trial had not closed early, nor received negative publicity. Dolutegravir is now widely available in routine practice, and children who did not receive this during the trial have been moved on to it Tenofovir alafenamide (TAF) is not currently available in routine care in a formulation suitable for young children. This means some participants in the TAF arm have been switched to the local standard of care
**P**rovider—who will provide the results to participants?	Participants in ICON8 had been followed up for between 5 to 8 years, with face-to-face follow-up visits at the clinic. Their main points of contact for the trial were the site staff (oncologist, research nurse and trial administrators)—they have not had direct contact with the clinical trials unit or sponsor. Some participants will have had the same staff throughout the trial, while others will have seen different site staff over the course of their follow-up. Some site staff have developed close relationships with participants (particularly at sites where there have no been many changes in staff working on the trial). The number of participants at each site who were alive at the time results were available varied from 1 to more than 50	Participants in CHAPAS-4 have been followed up for at least 2 years, but are no longer in follow-up, and have moved back to routine care rather than research clinics. Follow-up was done face-to-face. Their main point of contact with the trial was the clinical team at their site. The site teams for this trial have begun to disperse to other studies or roles, now the trial is over. Sites had large numbers of participants (ranging from 74 to 219) Site staff are the appropriate people to provide results to participants in CHAPAS-4, because they know the parents and participants, and this is in line with what the Participant Information Sheet told participants/parents/caregivers. Site staff are also best placed to answer any questions about the results from participants/parents/caregivers, knowing both the participant and the study
**E**xpertise and resources—what expertise and resources do you have access to for sharing results?	We had access to sufficient budget for printing and postage, but not to employ specialist filmmakers or graphic designers. We had access to expertise on the team on developing patient-facing communications tools and writing in plain language Sharing results with participants is included in CTU Standard Operating Procedures Sites were aware that they would be asked to share results with participants	We had budget available to cover the costs of translation, a graphic designer and printing The CHAPAS-4 trial has access to Youth Trial Boards who help develop materials that are suitable for children living with HIV. CHAPAS-4 also has access to expertise on the team on developing patient-facing communication tools and writing in plain language The activity is seen as important for CTU staff, and sites are aware of their role in sharing results with participants
**C**ommunication tools—which ones will you use?	We carried out patient and public involvement on the draft results summary to ensure it contained the information participants are likely to want to know. We provided the information in plain English and got input from patient representatives and ovarian cancer information specialists to ensure it was clear and easy to read We had access to people with some design skills in-house to allow us to format a clear printed summary and create the webpages. We were able to provide more than one way for participants to access results We used a combination of webpages (including links to further information and support, a short ‘talking heads’ style video alongside a plain language summary) and printed summary. We also offered an email option	We consulted with the Ugandan Youth Trial board over how to share results with participants. They recommended, in addition to meetings with participants and their families, developing an infographic or poster summarising the results graphically Once the results were known, we asked the Zimbabwe Youth Trial board to develop text summarising the results that will be of interest to participants and think about graphics for an infographic/poster. These were then passed on to a graphic designer to work on, along with translations of the text into the languages used for the Participant Information Sheet. Drafts of the posters were then shared with the Youth Trial Boards for comments, and these incorporated by the graphic designer. The posters are displayed at clinics and were shared during the participants meetings. Representatives from local ethics and regulatory bodies and local ART clinics where the children are being treated were also be invited. During the participants meetings, there was be a presentation of the results (using plain language) and other activities such as a short play by children to share their experiences of the trial. The meetings were also an opportunity to thank participants and their families and celebrate the achievements of the trial
**T**iming—when should results be communicated?	The results from ICON8 were unlikely to receive media coverage, given the disappointing nature of the results. The results did not have implications for the future treatment of participants. Participants were still in follow-up, but not frequently We were confident that the key message was unlikely to change during the peer review process. Given these factors, we felt it was appropriate to share results with participants after presentation and prior to publication. However, we did not want to delay sharing results until participants’ next clinic visit, as this may be several months away. We therefore decided to send the information on how to access the results by mail	The results were unlikely to receive media coverage in the mainstream media, being of more interest to specialist medical media. There are not immediate implications for participants’ treatment based on these results (most have already been switched to dolutegravir). This means it is not urgent to share results with participants, but we do not want to delay sharing results too long, as few staff will be available to share results if we leave it long. We are confident results are unlikely to change during the peer review process, so will share results between presentation and publication
Links to the interventions used	The Enhanced webpage can be found https://mrcctu.ucl.ac.uk/studies/all-studies/i/icon8/results-of-the-icon8-trial/ The printed summary can be found https://doi.org/10.1371/journal.pmed.1003798.s006 The Patient Update Information Sheet can be found https://doi.org/10.1371/journal.pmed.1003798.s005	An English version of the results poster (co-developed with Youth Trial Boards) can be found https://mrcctu.ucl.ac.uk/media/2489/chapas4results_english_a4_cmyk_3mmnobleed.pdf

## Discussion

### Summary of key findings

We propose several considerations when planning how to share results with participants in clinical trials. This includes how participants will be prepared and supported when receiving results and how the communication tool(s) will reach participants. Participant-related factors, such as demographics, education levels and computer literacy, alongside their health and expectations around receiving results, must also be considered. The trial results themselves (whether they will be considered as good, bad or neutral news by some or all participants, and their complexity) also need to be taken into account. Trials with more complex or potentially upsetting results (e.g. where the participants allocated to an arm did less well than participants allocated to other arms, or where one sub-group did less well than others) may need to offer participants additional support, for example through sharing results face-to-face or in individual video calls, or offering follow-up appointments or phone calls with doctors or research nurses if results are shared via written summaries. Trial results communication must also consider whether participants have developed relationships with site staff over the course of their participation, and how and from whom they are used to receiving communication about the trial. It may be appropriate to reflect this in some way, for example through allowing personalisation or one-to-one communication. The expertise and resources available to trial teams to communicate trial results is an important factor when deciding how this is done. Any communication tools used must reflect what the participants are likely to want to know and be understandable (using appropriate language(s) and reading levels) and accessible to the intended audience. It may be appropriate to provide a choice of tools, as different participants are likely to have different preferences and needs. The timing of when results are shared also needs to be carefully considered, avoiding participants finding out results from other sources prior to being informed by the trial team, if possible. Considering these factors, and involving patients and the public, can help develop communication tools and processes that are appropriate to the trial context, population and messages.

### Strengths of this study

A key strength of this study is its integration of qualitative data from both site staff and ICON8 trial participants, giving us insight into the views of those who are responsible for sharing results, alongside those who have experienced receiving trial results. Many of the site staff who took part in Show RESPECT worked across many trials and were able to draw on their experience from other studies in addition to Show RESPECT. The qualitative data provide a rich understanding of the perspectives of ICON8 trial participants and site staff on the experience of receiving or sharing trial results. Applying an established theoretical model (the Information Seeking and Communication Model [35, 36]) increased our ‘information power’ [37], through synthesising existing knowledge, extending the sources of knowledge beyond our empirical data and explaining relations between different aspects of the empirical data in a coherent way [37]. Applying the model helped us to ground our conclusions in the context of existing knowledge about the process of information seeking and communication.

Discussion of our findings with key stakeholders working on a wide variety of trials allowed us to ensure the framework is applicable beyond the ovarian cancer setting. The applicability of our framework to very different trials is illustrated by the example of the CHAPAS-4 trial, shown in Table 2. Applying the framework was helpful for thinking through how to share results with participants in CHAPAS-4. The answers to the individual questions were very different from those for ICON8, and very different communication approaches were selected, but the considerations were all relevant. While the framework does not directly prescribe how to share results, having a structured framework to follow gave confidence that nothing important had been overlooked. We envisage the framework being most useful as the basis of discussion of ideas and plans between members of the trial team and patient representatives.

### Limitations of this study

Show RESPECT was carried out within the context of a single trial, a limitation of this study, raising questions about the transferability of the findings to trials with different patient populations, diseases, results scenarios and settings. We acknowledge this possibility and emphasise that in this paper we focus on exploring factors that trialists should consider when preparing to share results with participants, rather than recommending that the approach that worked best within Show RESPECT should be used in trials with very different contexts or patient populations. We further acknowledge that we were not able to take account of ethnicity of respondents, nor on factors such as socio-economic background, as these data were not collected for this study. Very few of our patient participants reported having a first language other than English, but patient participants did report widely varying education levels.

The development of the framework was not a formal deliberative process—the framework is an output from our research that we believe will be of value to other researchers. However, we acknowledge that, given it is largely informed by evidence from a single trial, there may be considerations that we have missed that might be important for other trial contexts. We see this as being a starting point for improving practice in this area, but recognise that further refinement of the framework may be needed after it has been applied in a wide variety of trials. We invite readers to send us feedback around their experience of using the framework and will consider revising it in the future if further important considerations are identified or improvements need to be made.

### Our results in the context of what was already known

Our adaptable framework of factors to consider when planning how to share results is similar to guidance released by the UK Health Research Authority in 2023 [3], after the Show RESPECT patient results had been published. These similarities are unsurprising, given that the results of Show RESPECT helped inform this guidance, and several authors of this paper were involved in developing them. The HRA guidance on what to consider covers:

- Who will receive the findings
- How you will communicate the findings
- Giving participants a choice
- Responsibility for communicating findings
- Exceptions
- When to communicate findings
- Evaluating your communication [3]

Our findings around giving participants a choice over whether to receive the results or not reinforces previous recommendations that a two-stage approach should be used, offering results and then providing them, rather than simply distributing results to all participants [38]. Choosing not to access results was, for some patients, a way of protecting themselves from potentially finding out that they missed out on the better treatment. This concept of people choosing what information to engage with or not as a protective mechanism is similar to findings from the BRACELET study, where some bereaved parents of babies who died while participating in a trial for very high-risk neonates advised that communication from the trial should be managed in a way that would suit any parents who felt that they might be upsetting for themselves or their partner [39].

Only by providing information in a way that is understandable to the intended audience can we meet the objectives of sharing research results. Care needs to be taken when preparing results summaries, to ensure they are comprehensible for participants. Previous research has found that much written information about clinical trials exceeds the average reading age [40]. The UK National Health Service Digital Service Manual style guide states that they aim for a reading age of 9–11 years old where possible [41]. Artificial intelligence can be used to help researchers produce plain language summaries, but these will still require review from both investigators and patient representatives to ensure the content is correct and appropriate for the intended audience.

Many of our findings align with findings from the RECAP study [42, 43], supporting the transferability of our framework to trials beyond the UK ovarian cancer setting in which our work took place.

### Further research

Further research involving participants and site staff receiving and sharing trial results in trials with different patient populations, trial characteristics and results scenarios would be valuable for exploring the transferability of our findings to other contexts. Research is also needed to address how demographic factors such as geographical location, socio-economic status, ethnicity and different levels of language proficiency influence how results should be shared with participants.

## Conclusion

To ensure that trials meet their moral obligations to participants to share trial results, trialists must consider how results should be shared with participants from the planning stage of trials, to ensure that adequate resources are budgeted for and included in agreements with sites. Relevant information about how results will be shared should be included in the Patient Information Sheet. When deciding how to share results with participants, trialists should consider the following factors: how to support and prepare participants to receive results, including whether to use an opt-in or opt-out approach and who will be available to answer participant questions; how the results will reach participants; the characteristics and expectations of participants in relation to the results; what the results show and how they are likely to be perceived by participants; special considerations; who will provide the results to participants; the expertise and resources available for sharing results; the communication tool(s) to be used; and the timing of results communication. Patient and public involvement is essential for planning how to share results with participants, identifying the outcomes and study results that are important and relevant to participants, and developing the content of results summaries to ensure they are written in a clear and sensitive manner.

### Supplementary Information

Additional file 1: Description of methods. Text file describing the methods for this work (duplicated from our previous publications).

Additional file 2: SRQR checklist. Table showing where items from the SRQR checklist can be found in the manuscript.

Additional file 3: Characteristics of qualitative interviewees. Table showing the characteristics of qualitative interviews (this data has been previously published).

Additional file 4: The SHOW RESPECT adaptable framework concepts, and related themes, sub-themes and high-level codes from the Show RESPECT qualitative data. Table showing the concepts from the adaptable framework, and the themes, sub-themes and high-level codes that relate to those concepts.

Additional file 5: Illustration of the framework with findings from Show RESPECT. Qualitative findings from the Show RESPECT study that illustrate concepts from the SHOW RESPECT framework.

Additional file 6: Ethics approval letter. Letters from ethics committee confirming approval for the study.

## Data Availability

The protocol is available on the MRC CTU website https://www.mrcctu.ucl.ac.uk/media/1980/show-respect_protocol_v30_20aug2018_clean.pdf. The data that underlie the results reported in this article, after de-identification, will be available beginning 12 months after publication following the CTU’s standard moderated access approach (details of which are available https://www.mrcctu.ucl.ac.uk/our-research/other-research-policy/data-sharing/). Applicants will need to state the aims of any analyses and provide a methodologically sound proposal. Applications should be directed to mrcctu.datareleaserequest@ucl.ac.uk. Data requestors will need to sign a data access agreement at an institutional level.

## Abbreviations

BRACELET: Bereavement and Randomised Controlled Trials
CHAPAS-4: Children with HIV in Africa—Pharmacokinetics and Acceptability of Simple second-line antiretroviral regimens
CTU: Clinical trials unit
HIV: Human immunodeficiency virus
HRA: Health Research Authority
ICON8: An international phase III randomised trial of dose fractionated chemotherapy compared to standard three weekly chemotherapy, following immediate primary surgery or as part of delayed primary surgery, for women with newly diagnosed epithelial ovarian, fallopian tube or primary peritoneal cancer
ISCM: Information Seeking and Communication Model
PPI: Patient and public involvement
RECAP: REporting Clinical trial results Appropriately to Participants
Show RESPECT: Show Results to Participants Engaged in Clinical Trials
UK: United Kingdom
USA: United States of America
