# Supplementary material for: The SHOW RESPECT adaptable framework of considerations for planning how to share trial results with participants, based on qualitative findings from trial participants and site staff
Source: Trials. 2024 Jul 10;25:467. doi: 10.1186/s13063-024-08291-7 (PMC11234608; doi:10.1186/s13063-024-08291-7)
Supplement: Supplementary file 1 — Additional file 1: Description of methods. Text file describing the methods for this work (duplicated from our previous publications). [file 13063_2024_8291_MOESM1_ESM.docx]

# Additional File 1: Description of Show RESPECT methods

This information has been published previously (1-4).

## Patient and public involvement

Patient and public involvement (PPI) was integral to the design and conduct of this study, including through discussion groups, a PPI survey, patient representation on the study steering group, and input from patient groups and individuals on the design and content of the interventions, and interpretation of the results.

## Context of the ICON8 trial

The ICON8 trial is a phase III non-blinded randomised controlled trial looking at which chemotherapy schedule should be used for women with ovarian cancer (ISRCTN10356387). ICON8 compared three-weekly chemotherapy cycles (the current standard of care), to two different weekly schedules, with either both drugs being given weekly, or paclitaxel given weekly and carboplatin being given once every three weeks. Results from one of the co-primary endpoints, progression-free survival, were published in 2019, showing no difference in progression-free survival between the three arms(5).

## Qualitative data collection

The main source of qualitative data for Show RESPECT was semi-structured interviews with ICON8 patients and site staff who had been involved in sharing the ICON8 results with patients. Interviews were conducted either by telephone or face-to-face, in the participant’s own home or, for site staff, at the workplace where they conducted the trial. The mode of interview (telephone or face-to-face) was discussed on a telephone call prior to the interview, where time and date of the interview was also arranged, and questions about the study were answered. Interviews were guided by topic guides that were adapted as the study progressed to explore issues that emerged (for example, questions on sharing results with families of deceased participants were added to the topic guides based on issues that were raised by participants in early interviews). Interviews were audio recorded and transcribed.

In addition to the qualitative interviews, qualitative data were collected by free-text questions on the questionnaires that were completed by patients (after receiving results) and site staff (immediately after sharing results, and 2-3 months later). The questionnaires collected data on the views of participants and site staff on how the trial results were shared, including participant satisfaction and comprehension, and site staff views on feasibility, resources required, and acceptability. The free text questions were related to the quantitative questions, allowing the participants to explain why they had selected a certain response option, or raise issues that may be important to them. The topic guide, questionnaires, details of how these were administered, and researcher characteristics and reflexivity can be found in our previous publications(1, 2, 4, 6).

## Sampling and participants

We used a purposive sampling approach for the semi-structured interviews with both participants and site staff, allowing us to collect data from respondents with a range of characteristics that may be related to their experiences and views on sharing results. For ICON8 patients, this included age, education level, frequency of internet use and reported satisfaction with how the ICON8 results were shared, while for site staff this included their role, number of ICON8 patients at the hospital at which they work. For both groups, we included which interventions their hospital had been randomised to. Invitations to take part in interviews were sent out to ICON8 participants with the Show RESPECT questionnaire, with patients asked to complete a contact details form if they wanted to find out more about the interviews and to return it alongside their questionnaire. Invitations to site staff were sent by email. Respondents who expressed interest in the interviews and filled one or more gaps in the sampling frame were contacted by telephone with more information about the study. If they were willing to take part, a time and date was arranged for the interview. Interviews were carried out until all the gaps in the sampling frame were filled, or until no more volunteers were available who would fill a gap in the sampling frame. Using the Information Power model(7) to assess the necessary sample size, the study aim was reasonably narrow, focusing on just one aspect of trial experience (receiving or sharing results), although interviews did explore several approaches to results communication. The sample specificity was dense, with all interviewees having highly relevant experiences. As described in the analysis section below, an established model was applied during the analysis. The quality of dialogue in most interviews was strong, with good rapport established between interviewer and interviewee, resulting in a rich dataset. The analysis strategy was cross-case, examining data from different participants for common patterns and ideas. Taken together, these factors suggested that a moderate sample size of around 20 participants would provide sufficient information power to meet the study aims.

## Qualitative analysis

We used a reflexive thematic analysis approach(8), with a critical realist stance (taking the ontological position that an external reality exists that is independent of our beliefs and understanding, but that our knowledge of that external reality is influenced by our historical, social and cultural situation(9)), to analyse the data. The first stage was familiarization with the data, which included reading the transcripts while listening to the audio recordings of the interviews. AS coded transcripts using a combination of inductive codes (creating labels for sections of data that evoke important features of the data that might be relevant to the research question(10)) and deductive codes (labels applied to sections of data based on *a priori* concepts in the topic guide and research questions), within the software Atlas.ti version 8.4. Sections of the data were given as many codes as were appropriate to cover the content of the section. Initial codes were then grouped into potential themes and sub-themes, which were discussed with staff from the ICON8 and Show RESPECT trial management teams. At this stage, we identified that the Information Seeking and Communication Model (ISCM)(11, 12) fitted the data well as a framework for high-level categorisation and conceptualization, so we mapped our potential themes, sub-themes and codes using concepts from the model. The model covers the perspective of both the Information User (trial participants) and Information Provider (site staff), incorporating:

- the communication process
- communication medium
- information product
- the context in which the Information User and Information Provider are operating
- what the Information User does to process that information
- the outcomes from receiving that information.

Once coding of all the interview transcripts was complete, we then coded the free-text responses from the questionnaires using the same coding scheme as the interview data, with additional codes added where necessary. As analysis progressed, we refined codes, themes, and sub-themes, creating network diagrams to explore linkages between codes within each candidate theme and sub-theme. AS read and compared quotations linked to all the codes within the network to check for consistency of meaning for each code and created links between related codes (such as one code being associated with another, being an example of something described by another, higher-level code, or being the opposite of or contradicting another code). Where a theme or sub-theme included data from both site staff and patients, we looked at the quotations by type of respondent, to see how their perspectives compared.

Participant checking (returning data or results to participants for checking) did not take place, but a patient and public involvement discussion group and discussion groups with site staff were held to reflect on the preliminary findings and interpretation.

## References

1. South A, Joharatnam-Hogan N, Purvis C, James EC, Diaz-Montana C, Cragg WJ, et al. Testing approaches to sharing trial results with participants: The Show RESPECT cluster randomised, factorial, mixed methods trial. PLoS Medicine. 2021;18(10).

2. South A, Bailey J, Bierer BE, Burnett E, Cragg WJ, Diaz-Montana C, et al. Site staff perspectives on communicating trial results to participants: Cost and feasibility results from the Show RESPECT cluster randomised, factorial, mixed-methods trial. Clinical Trials. 2023:17407745231186088.

3. Show RESPECT team. Show RESPECT: Show RESults to Participants Engaged in Clinical Trials: A cluster randomised factorial trial of different modes of communicating results to participants of the ICON8 phase III ovarian cancer trial London2018 [cited 2021. Available from: <https://www.mrcctu.ucl.ac.uk/media/1980/show-respect_protocol_v30_20aug2018_clean.pdf>.

4. South A. Showing RESPECT: a mixed methods study into communicating the results of a Phase III clinical trial to trial participants. London: UCL; 2023.

5. Clamp AR, James EC, McNeish IA, Dean A, Kim JW, O'Donnell DM, et al. Weekly dose-dense chemotherapy in first-line epithelial ovarian, fallopian tube, or primary peritoneal carcinoma treatment (ICON8): primary progression free survival analysis results from a GCIG phase 3 randomised controlled trial. Lancet. 2019;394(10214):2084-95.

6. South A. Show RESPECT: OSF; 2023 [Available from: <https://osf.io/6tpf4/?view_only=10add9b2f1b34814a400e329988d993d>.

7. Malterud K, Siersma VD, Guassora AD. Sample Size in Qualitative Interview Studies: Guided by Information Power. Qualitative Health Research. 2015;26(13):1753-60.

8. Braun V, Clarke V. Reflecting on reflexive thematic analysis. Qualitative Research in Sport, Exercise and Health. 2019;11(4):589-97.

9. Archer M, Decoteau C, Gorski P, Little D, Porpora D, Rutzou T, et al. What is Critical Realism? Perspectives: A Newsletter of the AS Theory Section. 2016.

10. Braun V, Clarke V. The Reflexive TA process Auckland, New Zealand: The University of Auckland; [Available from: <https://www.thematicanalysis.net/doing-reflexive-ta/>.

11. Robson A. Modelling information behaviour: linking information seeking and communication [Ph.D.]. Ann Arbor: The City University (London) (United Kingdom); 2013.

12. Robson A, Robinson L. The Information Seeking and Communication Model. J Doc. 2015;71(5):1043-69.
