# Supplementary material for: The SHOW RESPECT adaptable framework of considerations for planning how to share trial results with participants, based on qualitative findings from trial participants and site staff
Source: Trials. 2024 Jul 10;25:467. doi: 10.1186/s13063-024-08291-7 (PMC11234608; doi:10.1186/s13063-024-08291-7)
Supplement: Supplementary file 3 — Additional file 3: Characteristics of qualitative interviewees. Table showing the characteristics of qualitative interviews (this data has been previously published). [file 13063_2024_8291_MOESM3_ESM.docx]

### Additional File 3: Characteristics of qualitative interviewees

These results have been previously published (26, 27).

| **Characteristics** | **No. of interviewees** |
| --- | --- |
| **Patient Interviewees** | **13** |
| **Interventions offered**^[[1]](#footnote-1)^ | |
| Basic Webpage | 8 |
| Enhanced Webpage | 5 |
| Mailed Printed Summary | 6 |
| No Mailed Printed Summary | 7 |
| Email List Invitation | 9 |
| No Email List Invitation | 4 |
| **Interventions used**^1^ | |
| Basic Webpage | 5 |
| Enhanced Webpage | 2 |
| Mailed Printed Summary | 6 |
| Opted out of Mailed Printed Summary | 0 |
| Email list | 0 |
| Had not found out the results prior to interview | 2 |
| **Reported satisfaction with how the results were shared (from quantitative questionnaire)**^[[2]](#footnote-2)^ | |
| Very unsatisfied, quite unsatisfied or neither satisfied nor unsatisfied | 5 |
| Quite satisfied or very satisfied | 5 |
| **Reported highest level of education**^[[3]](#footnote-3)^ | |
| A levels or lower | 6 |
| Degree or higher | 6 |
| **Reported frequency of internet/email use** | |
| Less than once a week | 2 |
| More than once a week | 11 |
| **ICON8 randomised allocation** | |
| Three-weekly chemotherapy (control arm) | 3 |
| Weekly chemotherapy (ICON8 intervention arms) | 10 |
| **Age group** |  |
| ≤50 | 0 |
| 51-60 | 2 |
| 61-70 | 6 |
| ≥71 | 5 |
| **Site staff interviewees** | **11** |
| **Show RESPECT randomisation^[[4]](#footnote-4)^** |  |
| Works at site randomised to printed summaries | 6 |
| Works at site not randomised to printed summaries | 6 |
| **Site strata (based on number of ICON8 participants)^4^** |  |
| Small | 2 |
| Medium | 5 |
| Large | 5 |
| **Job role** |  |
| Oncologist | 2 |
| Nursing | 5 |
| Administrative | 4 |

1. Adds up to >13 as some participants were offered more than one intervention [↑](#footnote-ref-1)
2. Data missing from 3 participants’ questionnaires [↑](#footnote-ref-2)
3. Data missing from 1 participant’s questionnaire [↑](#footnote-ref-3)
4. One interviewee worked at two sites of different sizes, randomised to different interventions [↑](#footnote-ref-4)
