# Supplementary material for: The SHOW RESPECT adaptable framework of considerations for planning how to share trial results with participants, based on qualitative findings from trial participants and site staff
Source: Trials. 2024 Jul 10;25:467. doi: 10.1186/s13063-024-08291-7 (PMC11234608; doi:10.1186/s13063-024-08291-7)
Supplement: Supplementary file 4 — Additional file 4: The SHOW RESPECT adaptable framework concepts, and related themes, sub-themes and high-level codes from the Show RESPECT qualitative data. Table showing the concepts from the adaptable framework, and the themes, sub-themes and high-level codes that relate to those concepts. [file 13063_2024_8291_MOESM4_ESM.docx]

# Additional File 4: The SHOW RESPECT adaptable framework concepts, and related themes, sub-themes, and high-level codes from the Show RESPECT qualitative data

| **Show RESPECT Framework item** | **Theme** | **Sub-themes** | **High-level codes** |
| --- | --- | --- | --- |
| **S**upporting and preparing trial participants to receive results | Patients’ desire to  receive trial results | Wanting to know the results | Demand for results |
|  |  |  | Closure |
|  |  |  | Seeing if the trial was any use |
|  |  |  | We do trials to find out the results |
|  |  | Not wanting to know the results | Not wanting to think about cancer |
|  | Patient reactions  to finding out the  ICON8 results | Intellectual responses | The results are interesting |
|  |  |  | The results are important |
|  |  |  | Patients’ interpretations of the results and their implications |
|  |  |  | Raising questions |
|  |  | Emotional responses | Surprise |
|  |  |  | Positive emotional responses |
|  |  |  | Negative emotional responses |
|  |  |  | Mixed emotional responses |
|  |  |  | Reflections on the results and their randomised treatment allocation |
|  | Opt-in vs opt-out  approaches | Opt-in approaches | Timing of opt-in |
|  |  |  | Feasibility of opt-in approaches |
|  |  | Opt-out approaches | Concerns with opt-out |
|  |  |  | Opt-out approaches are good |
|  | Patients’ understanding of potential trial outcomes |  | Understanding potential trial outcomes |
|  |  |  | Understanding their prognosis |
|  |  |  | Lack of equipoise at time of joining trial |
|  | Patients’ access to support |  | Sources of support |
| **HO**w will the communication tool(s) reach participants? | Views of patients  and site staff on  the communication  medium | Ease of access | Accessibility of posted printed summaries |
|  |  |  | Not knowing how to access the results |
|  |  |  | Accessibility of electronic approaches to sharing results |
|  |  |  | Other ways in which patients found out the ICON8 results |
|  |  | Approaches based on a printed summary | Accessible to all |
|  |  |  | Good for sharing |
|  |  |  | Cost |
|  |  | Electronic means of communication | Access to computers / internet |
|  |  |  | Emails are quick and simple |
|  |  |  | Emails are more personal |
|  |  |  | Feasibility of emails |
|  |  | Personal approaches | Telephone calls |
|  |  |  | Face-to-face |
|  |  | Group meetings | Feasibility |
|  |  |  | Can clarify things |
|  |  |  | Not private |
|  |  | Giving participants choice | Need for different approach in different circumstances |
|  |  |  | Offer variety |
|  | The process of sharing results |  | Checking participants’ health |
|  |  |  | Finding addresses |
|  |  |  | Sending out the Patient Update Information Sheet |
|  |  |  | Leaving time between the stages of sharing results |
|  |  |  | Sending out Printed Summaries |
|  |  |  | Further follow-up and support |
| **W**ho are the trial participants? | Disease and outcomes of interest |  | Prognosis of patients |
|  | Demographic factors |  | Age |
|  |  |  | Education level |
|  |  |  | Frequency of internet and email use |
|  | Health factors |  | Randomised arm |
|  |  |  | Health at the time results are shared |
|  |  |  | Experience of side-effects during the trial |
|  | Patients’ expectations around receiving results |  | Expecting to receive results |
|  |  |  | Not expecting to receive results |
|  | Patients’ reflections on being part of a trial |  | Cancer experience |
|  | Patients’ experiences of receiving the ICON8 results | Reading and processing the results | Reading the results |
|  |  |  | Processing the results |
|  |  |  | Reading the results with others |
|  |  | Outcomes of finding out the ICON8 results | Discussing the results with others |
|  |  |  | Keeping the results |
|  |  |  | Sharing the results and their trial experiences with others |
| **RE**sults- what do they show? | Trial design, intervention(s) and control |  | Phase of trial |
|  |  |  | Placebo-controlled trials |
|  | What the trial results show |  | (No) need for different approach if there is a large difference |
|  |  |  | Easier to share ‘good’ news |
|  | Concerns |  | Depends on what’s being communicated |
|  |  |  | Emotional impact of the results |
| **S**pecial considerations | Which trials should share results with  participants? |  | Duty of candour |
|  |  |  | Sharing results – every trial |
|  |  |  | Sharing results - exceptions |
|  |  |  | Trials in emergency settings |
| **P**rovider – who will provide the results to participants? | Relationship between site staff and participants |  | Close relationship |
|  |  |  | Not close relationship |
|  | Finding out the results in other ways |  | Being told results by site staff |
|  |  |  | Researching it for themselves |
|  | Personalisation | (No) need for personalisation | Need for personalisation |
|  |  |  | No need for personalisation |
|  |  | Personalising the interventions | Cover notes |
|  |  |  | Telephone calls |
|  | Site staff’s access to and experience of sharing previous trial results | Site staff’s access to study results | Access to study results |
|  |  | Experience of sharing trial results with participants in other trials | Experience from other trials |
|  |  | Support from trials units to share results in other trials | Support from CTU |
|  | Responsibility for sharing results with participants |  | Should come from Sponsor |
|  |  |  | Should come from site |
| **E**xpertise and resources – what expertise and resources do you have access to for sharing results? | What inhibits site staff from sharing trial results with participants? | Concerns: practicalities |  |
|  | Challenges |  | Time |
|  |  |  | Working out who to send results to |
|  |  |  | Giving patients options |
|  |  |  | Patients not receiving posted documents |
|  |  |  | No challenges |
|  |  |  | Resource implications |
| **C**ommunication tools – which ones will you use? | Views on Show RESPECT interventions | Layout and structure | Headings and broken-up text |
|  |  |  | Use of columns |
|  |  |  | Use of colour |
|  |  |  | Structure |
|  |  |  | Navigation |
|  |  |  | Text size |
|  |  |  | Paper |
|  |  | Preferences between the interventions | Preferred webpage |
|  |  |  | Preferred printed summary |
|  | Information | Understandability of the information | Easy to understand |
|  |  |  | Difficult to understand |
|  |  | Level and length of information | Short and simple |
|  |  |  | Too wordy |
|  |  | Language used | Clear and easy |
|  |  |  | Scientific terminology |
|  |  | Information items | Interesting and important items |
|  |  |  | Unnecessary information |
|  |  |  | Missing information |
|  |  |  | Information on survival |
|  |  |  | Diagrams |
|  |  |  | Links to further information and support |
|  |  |  | Frequently asked questions |
|  |  |  | Video |
|  |  |  | Thanks |
|  |  |  | Other information |
| **T**iming – when should results be communicated? | Timing of sharing results |  | Timing of communicating results |
