# Supplementary material for: The SHOW RESPECT adaptable framework of considerations for planning how to share trial results with participants, based on qualitative findings from trial participants and site staff
Source: Trials. 2024 Jul 10;25:467. doi: 10.1186/s13063-024-08291-7 (PMC11234608; doi:10.1186/s13063-024-08291-7)
Supplement: Supplementary file 5 — Additional file 5: Illustration of the framework with findings from Show RESPECT. Qualitative findings from the Show RESPECT study that illustrate concepts from the SHOW RESPECT framework. [file 13063_2024_8291_MOESM5_ESM.docx]

# Additional File 5: Illustration of the framework with findings from Show RESPECT

These results have been previously published as part of a doctoral thesis [1].

Contents

[Supporting and preparing trial participants to receive results 2](#_Toc154043730)

[How will you prepare participants for receiving results, and give them the opportunity to opt-in or opt-out? 2](#_Toc154043731)

[How will you provide support to patients who have additional questions or are distressed by the results? 2](#_Toc154043732)

[What other support is available to them to help them understand the results, or deal with them emotionally? 4](#_Toc154043733)

[How will the communication tool(s) reach participants? 4](#_Toc154043734)

[Who are the trial participants? 6](#_Toc154043735)

[What are the demographic characteristics of your trial participants? 6](#_Toc154043736)

[How well are your participants likely to be? 7](#_Toc154043737)

[What expectations do your participants have around receiving trial results? 9](#_Toc154043738)

[What will participants want to do with the results? 10](#_Toc154043739)

[Results – what do they show? 11](#_Toc154043740)

[What is the disease area and outcome of interest? 11](#_Toc154043741)

[What do your trial results show, and how complex are they? 12](#_Toc154043742)

[What is your trial design, intervention(s) and control? 15](#_Toc154043743)

[Special considerations 16](#_Toc154043744)

[Provider - who will provide results to participants? 16](#_Toc154043745)

[How close are relationships between site staff and participants likely to be? 18](#_Toc154043746)

[How many participants do sites have? 19](#_Toc154043747)

[Expertise and resources – what expertise and resources do you have access to for sharing results? 20](#_Toc154043748)

[Communication tools – which ones will you use? 20](#_Toc154043749)

[Timing - when should results be shared with participants? 21](#_Toc154043750)

[References 22](#_Toc154043751)

# Supporting and preparing trial participants to receive results

## How will you prepare participants for receiving results, and give them the opportunity to opt-in or opt-out?

Consideration should be given to how participants will be prepared to receive results and given the opportunity to opt-in or opt out. Some trials may have done this as part of the informed consent process. Where this is the case, thought needs to be given to whether this needs to be checked with participants when results are available, giving them the opportunity to change their mind.

*"I think it would be a really good idea going forward to, you know, ask patients if they want the results when consenting to the clinical trial, And, again, ask them once they’ve completed the treatment if they’d still like to receive the results."* HLRNI03: Research Nurse, large site

The other driver behind discussion of the need to prepare participants prior to sharing results was that results may potentially be upsetting, depending on what the trial found, and the arm the patient had been randomised to.

*"I think if you put it on the update sheet that there was… not saying which particular arm was the best, but just saying that they should be prepared for some sort of news that they may not find good. I don’t know, but just preparing them beforehand might or would help if that were the case."* CLTCI04: Trial Coordinator, large site

## How will you provide support to patients who have additional questions or are distressed by the results?

Some participants may require further support or have questions about the results. Making sure participants know how to access that support or information is important, whether that is from site staff, an existing patient helpline, or other sources.

*"That was the thing, I think, we were slightly concerned about was, well, what if that raises questions, which again is why we put that you know, compliments slip in… you know, do phone us if you’ve got any issues with it or queries or anything."* DMRNI02: Research Nurse, medium site

Having a good relationship with their research nurse or oncologist meant that patients felt able to ask questions about the results, if needed.

*"I suppose to some extent, it’s on the research nurses because the two that I saw are really great. I get on really well with them and I don’t feel afraid to ask them any questions. But I think that’s more a personal thing really. It’s quite difficult for somebody if they can’t relate to the people they’re seeing, for whatever reason, obviously the next best option would be to have some paper to take away and read in their own time."* DMI01: Patient, medium site, close relationship to site staff

However, some patients did not want to bother busy site staff with questions (at least until they were scheduled to see them for a routine check-up), so had less opportunity to ask questions or seek clarification, and may have forgotten their questions by the time their scheduled visit arrived. In that situation, having alternative routes to access support or ask questions (such as the FAQ section and links to support on the enhanced webpage/email) might be particularly useful.

*“I do have numbers to ring the nurses at the [BL Hospital], but you never really want to bother them.”* BLI01: Patient, large site

*"I think by the time they had it in the post and they came back to clinic, they’d forgotten all about it really and that’s probably why they didn't speak to the PI [Principal Investigator]."* AMRNI05: Research Nurse, medium site

### What other support is available to them to help them understand the results, or deal with them emotionally?

The patients interviewed varied in terms of how much support they had access to, from family and friends they could talk to about their health, and patient support groups. Some patients and site staff felt that features like links to further information and support and ways of submitting questions may be particularly valuable for patients with less access to support, or who feel less able to ask their site staff questions.

*"The only real feedback I had was from some of the written in [E Hospital] with that patients just* *were a little bit shocked it wasn’t better prognosis all around. Just not having anyone to turn to at* *the time when it landed on her letterbox because she lived alone. So, all those patient factors are* *really important whether they’ve got someone with them when they’re going through. Just like* *opening exam results, you initially need someone with you."* EBLMCLI02: Oncologist, large and medium sites

# How will the communication tool(s) reach participants?

In Show RESPECT, electronic communication media (the webpages and email list) were preferred by some participants because of their speed, and by some site staff because of their ease of delivery. Many site staff and patients had concerns around using electronic means of communication, as they felt that many ICON8 participants do not have access to computers, email or internet. Where electronic means were preferred, this was often in combination with a printed summary for those who are unable to access the internet.

*"Sending an email like this to the participants would be a really good idea for those who ask for the results. I think that would be really useful. Again we could just forward this email onto them quite easily. And so I think that would be a really quick, you know, and simple way of giving the results by doing that. So, I think that’s a really good idea."* HLRNI03: Research nurse, large site

*"I know quite a few that wouldn't bother and don't like things online anyway... I hate to say it but even my age group don't like getting things on email. They like it in their hand."* BMI02: Patient, medium site, aged 71 or older

Several patients commented that they would have liked to have received results in a more personal way, through face-to-face or telephone conversation with their research nurse or consultant. For some it was about the opportunity for discussion and explanation, or the chance to hear what their research nurse or consultant thought about the results. Others said that they would feel more valued if they received the results in a more personal way. Some patients felt that finding out the results directly from site staff would be preferable to reading the information, as site staff would be able to make them more palatable. Some site staff felt uncomfortable not being able to gauge participants' reaction, and whether they needed further support. When talking about personal approaches to sharing results, several patients raised the resource implications, leading some to conclude that the drawbacks outweighed the benefits of more personal communication.

*"When you see it in black and white it’s really final, isn’t it. Whereas a health professional might be able to sugar the pill a bit."* GSI01: Patient, small site, who had not wanted to find out the results

*"I wouldn't have minded face-to-face, I suppose if you’ve got questions that’s useful but it’s a lot of waste of NHS money and time, when they can just send you something that you can read yourself."* BMI01: Patient, medium site

Patients and site staff recognised that different patients have different preferences and needs when it comes to receiving trial results, and many recommended that researchers should give patients options on how to receive results, allowing patients to pick the option that best meets their needs.

# Who are the trial participants?

## What are the demographic characteristics of your trial participants?

Site staff felt that the age of participants would affect how they would prefer to receive the results. They hypothesised that older patients would prefer printed summaries, while younger patients may be happier with webpages or emails. This distinction may reduce over time, as people who are familiar with computers and the internet get older.

*"I think you’ve got to look at the age group of the patients. I think that is the main thing. You’ve got to look at the age group of the patient. So everybody is individual, so like, if you are looking at maybe 65 and above, they would mostly prefer written summaries. Whereas the younger group will want the website."* BMRNI04 – Research Nurse, medium site

The patient interviewees who did not have at least A level qualifications seemed less satisfied, struggling to understand the results, or to access them. Some site staff felt that more educated participants may want more detail than participants with fewer qualifications, however from the participant interviews this was not necessarily the case, with some highly educated participants wanting only headline results, while others wanted a lot of detail.

In the qualitative interviews, participants' frequency of using email/internet did not seem to affect satisfaction. Some participants with lower computer literacy got the help of family members to access results (or asked site staff for printed copies), which may have mitigated the challenges for those who were randomised to no printed summary. Patients’ views on whether the results were easy to access did not vary by randomisation, suggesting online approaches were not inaccessible to most people. However, many site staff strongly preferred the printed summary, as they felt it was accessible to all, unlike approaches relying on access to internet/email.

*"The web page is okay but when I look at all the participants that we have, there is only one who uses the internet. The rest are old school. They prefer face-to-face or written."* BMRNI04 – Research Nurse, medium site

## How well are your participants likely to be?

Factors related to participants’ health, and their experience while taking part in the trial, may also affect how trial results should be communicated. One patient talked about email being fine for patients who were doing well, but perhaps being less appropriate for patients whose health was poorer, or who had less access to support.

CLI01: *“It would have been fine for me but it may not have been fine for other people. Not everybody has survived this as well as I have.”*

IV So your health status makes a difference to receiving this information?

CLI01: *“Yes. So, yes, I could receive that by email without a problem. Somebody else might have found that more difficult”* CLI01: Patient, large site

This desire for different modes of communication to those in the arm with poorer performance overall seemed particularly important if the patients themselves had experienced disease progression.

*"I think I would have liked the doctors to talk to me about that. If there really was a finding that actually, people were living longer and I’d got secondaries or something, yes, I would have liked to have been spoken to about that rather than finding out on the website."* CLI01: Patient, large site

Site staff also felt that more care was needed when sharing results with participants who were in poor health at the time results are available to share.

*"I think you’d probably have to be a bit more careful in terms of sharing results with patients who were very unwell and closer to their end of life. Particularly if they’ve reached the point where they are, I suppose, have come to terms with the terminal nature of their illness. Sharing information that might bring back difficult memories at that point, might be more difficult. I think it’s probably still best practice that if we do have that information available and we’re seeing the patient, that we ask them whether they want to know about the outcome of the trial."* HLCLI02: Oncologist, large site

There did not seem to be a relationship between the severity of side-effects patients experienced during their trial treatment, and their satisfaction with how the results were shared. Those with side-effects seemed to find it comforting to know others had had similar problems.

## What expectations do your participants have around receiving trial results?

Participants had differing expectations around whether they would receive the results. Among those who had not been expecting to receive the results, some had assumed that, as they were still in follow-up, results would not be available yet. Others had assumed that they would have died before the results were available. Some had put it out of their mind, while others remembered being explicitly told by their doctors that they would not be told the results. One interviewee talked about previously having participated in paid drug trials, and having not received results from that, did not expect results from ICON8.

*"I assumed that I would never know the results, that it would be… Well, first of all, I thought well I’ll probably be dead anyway, but no, I didn’t think they would be available. I thought trials probably went on for much longer, and that they would wait until people died before they assessed it."* GSI01: Patient, small site

There was a sense among some site staff that participants' expectations around receiving results had shifted in recent years, with participants now being more likely to expect to receive them. This change in expectations may be because there is more discussion of the issue at the time participants join the trial.

*"It’s something that I think is becoming more important. A lot of our patients are becoming more empowered. They’re wanting to seek more information. Treatment of cancer is becoming more complex, often patients will survive for longer and live with their cancer as a chronic illness. Probably there are more trial participants who are keen and interested in finding out the results of studies that they have taken part in, in the past. It is becoming a greater priority for us to engage with them in this setting."* HLCLI02: Oncologist, large site

## What will participants want to do with the results?

Once patients had received the results, some then discussed those results with others, including family members and friends, although these discussions may not have been in depth. Some patients had not discussed the results with friends or family, as they thought others would not understand, or be interested.

Many of the patient interviewees kept folders containing all the information they had received about the trial and their cancer treatment. In this context, patients appreciated having the printed summary that could be easily added to their files. Some women said if they had not received the printed summary, they would have printed out the information from the webpage, to allow them to file it for future reference.

*"It was easy to read over a period of time and I could keep a copy without finding a printer."* BMQ05: Patient, medium site

Some of the women I spoke to were keen to share their experience of cancer treatment and being on a trial with other cancer patients, or share the results of the ICON8 trial with other patients.

# Results – what do they show?

## What is the disease area and outcome of interest?

Ovarian cancer is a serious condition, often with a poor prognosis. The ICON8 trial aimed to improve progression free survival (the time until the disease gets worse or the patient dies) and overall survival. This means participants had been very invested in the success of the trial, with strong motivation to hope that the trial could improve outcomes for themselves personally, and for future patients.

One participant who did not want to find out the trial results for ICON8 said she would have wanted to find out the results if she had been taking part in a trial for a less serious condition.

IV: If it had been a trial looking at how to treat your heel problems, that might have been different?"

GSI01: *"Oh, that’s fine because it’s a heel, I wasn’t going to die of that. It might have been a nuisance, I might have moaned about it a huge amount, but that was a different matter; that wasn’t life or death."* (Patient, small site, did not want to receive results)

Site staff felt that the severity of the disease, and survival being an outcome, meant that extra care needed to be taken when sharing results. By the time the first results were available a substantial number of participants had died. Show RESPECT was looking at communication of results to participants who were alive at the time results were available, but the ICON8 results inevitably reflect that some participants were not so fortunate. Site staff felt this may be sobering or upsetting to some patients.

*"If you were alive, you read the results, and there was a high mortality rate, I don’t know how that would make me feel. Depressed, and grateful that I was still there to read the results"* GMTCI02: Trial Coordinator, medium site

However, the severity of the disease may not necessarily make a difference to how results should be communicated, if participants are aware of their prognosis at the time they join the trial. The patients interviewed did seem aware of the severity of ovarian cancer, although some sought to avoid information on this in order to protect themselves, and one oncologist described this understanding as something that grows over time, rather than necessarily being fully understood at the point of diagnosis, when they are invited to join the trial.

*"Because most of these patients, when they were recruited into the study, they know and they were told that some of them will be stage IIIc ovarian cancer, which they know how bad their chances are. So no, I don't think it affects that. They sort of understand. Patients understand, especially how far their stage is and what it involves."* BMRNI04: Research nurse, medium site

## What do your trial results show, and how complex are they?

ICON8 found no difference between the three arms, in terms of progression-free survival, with little difference in side-effects. As such, there were no clear 'winners' or 'losers' from taking part in the trial. This may make receiving the results a less emotionally intense experience for participants. Some trial results may also be harder to understand than others (e.g. where the results are uncertain, or when the effect of an intervention varies by sub-groups).

Doctors may be more keen to share results with participants if the results are seen as 'good'. Sharing results may be more difficult in certain result scenarios (for example if the intervention caused harm). This was not seen as a reason not to share results, but needs to be taken into account when deciding how to share results.

*"I think, when there’s some really good results a doctor always feels that’s what they want to tell their patients. Whereas if there’s a marginal benefit, then you’re likely to not really want to say too much of the results."* EBLMCLI02: Oncologist, large and medium sites

Some women felt that their emotional response would have been different, had the results showed a difference between the arms. Patients speculated that they may have felt angry or upset if their treatment arm turned out to be less good, but the extent of this may depend on their own health at the time of receiving results, as well as the results for the group overall. Some participants and site staff felt that if the results had been different (complex or potentially upsetting), it may be better to communicate them in a different way, with more personal approaches generally being preferred, giving patients more support to process that information, while some patients would want less information. Site staff felt it was important to know what arm a participant was on before telling them the results, if there was a difference between the arms, in order to share the results carefully and sensitively.

*"It would depend if it raised more questions perhaps. So maybe a clinician would have been better suited I suppose, if it was going to have that effect. Maybe the clinician giving a paper and discussing it in clinic maybe better for them than obviously reading it at home on their own."* GSTCI03: Trial coordinator, small site

*“If it went into my head that I was going to see more bad news about my participating group I might be less inclined to want to see a written report and just a referral to a website. Because this is almost... When you receive this [printed summary] you have to look at it whereas with the website you may think, oh, I’m not going to bother. You can ignore it more easily if you feel that your group is not going to have any more good news or better results.”* BLI01: Patient, large site

However, not all patients and site staff felt that, had the results been different, they would have wanted to receive/share the results in a different way. One patient, whose disease had progressed, talked about it not mattering to her too much, as she had made the decision to join the trial in the knowledge that it might help her, but it might not. She preferred not to dwell too much on what might have been, focusing instead on the future. While how the results are shared may not need to change, depending on the results, the way it is written needs to be carefully thought through.

*"It would be the same. Of course, maybe if they had been in an arm which had been very inferior, very different, you would feel a little bit disappointed for them especially if the cancer is coming back. You know? But I mean... I don't think there would be a difference really. Because what is done has been done. You can’t undo it."* BMRNI04: Research nurse, medium site

While some site staff would want to give the results face-to-face in clinic, patients may require more time and privacy for processing potentially distressing information, if they were on the inferior arm, which the clinic setting may not necessarily offer. This led other site staff to prefer giving patients the information to access in their own time and space, via a webpage or printed summary, with the offer of further support if needed.

*"I think if the results had been bad (in terms of I would have had to tell them that this treatment arm is better than your treatment arm), I think how they react to that, the clinic isn’t as private as you would want. If I had to tell them to their face I don’t think it would have been as good as me just sending them something on the web page and then putting at the bottom, you know, they needed any further support or whatever they can just call me. Instead of having them be in front of everyone reacting to it, be able to read it on their own time in their own space and react how they would want to react."* CLTCI04: Trial Coordinator, large site

## What is your trial design, intervention(s) and control?

ICON8 was an open-label phase III trial comparing different dosing schedules for standard chemotherapy drugs, rather than testing a new drug. Many patients saw the trial as low-risk when they joined, as the chemotherapy drugs being used were well established, with only the frequency and dose varying between arms. Placebo-controlled trials were seen to raise more complex issues around communication of results, such as the practicalities of unblinding, and some participants not having received an active treatment. Sites and participants do not always find out which arm participants had been randomised to in placebo-controlled trials, and if they do it is often long after the trial has finished. Patients may have wanted to find out the results more personally, through a conversation with their doctor, had the trial been placebo controlled. Site staff felt that earlier phase trials might raise different issues to phase III trials, and it may not always be appropriate to share results with participants in early phase trials, as results may be harder for patients to interpret as these trials do not focus efficacy. Another type of trial where site staff felt it might be more difficult to share results is in trials in emergency settings, where patients are not asked for informed consent prior to being randomised.

# Special considerations

ICON8 had not closed early or received negative publicity during the course of the trial. However the results conflict with those from a previous study (conducted in Japan), which participants had been informed about in the Patient Information Sheet. This affected some people’s expectations about what the trial would find, and the difference required some acknowledgement in the results summary.

*“I was [surprised] actually because I thought that the reason this was being done was because the Japanese women had found it easier and had found it better to have a more gentle approach. And I saw from this that it wasn’t only the UK, there were other hospitals throughout the world, so it might be something to do with the Japanese diet or their way of life, their whatever, it could be a lot of environmental factors. So, yes, it did surprise me.”* CLI01 – patient, large site

# Provider - who will provide results to participants?

There were differing views around who should be responsible for sharing results with participants. The most common view was that it should come from the site that had been looking after the patient, rather than the trial Sponsor. Reasons for this include that it is the site rather than the Sponsor that has the relationship with the participant, and is in a position to support the participant, and that the logistics of the Sponsor sending out the results would be impractical. Conversely, a research nurse at a site that had a large number of participants in trials was concerned that, if instituted for all trials, this would be extremely time-consuming for site staff, and said it would be better for the results to go directly from Sponsors to the participants.

*"I think it’s probably a responsibility for us as investigators to, when we’ve got patients who are keen to participate in trials, to be open and keep them informed in terms of the outcomes of the studies that they have been participating in, if they want to be informed about that."* HLCLI01: Oncologist, large site

*"The only problem with our site is we recruit so many patients onto our trials, it would be extremely time consuming. Now whether we could, you know, whether the Sponsors could send something out directly to the patients themselves, you know, with an option to receive the results or not. That would probably be a better option, you know, I don’t know. Again, it’s just the volume of patients that we’ve got on trial here. So, it’s a, you know, it’s a lot of information to send out to people, you know, when we’ve got so many patients on trial."* HLRNI03: Research Nurse, large site

Understanding of what was happening in terms of patients’ treatment was important for sharing results. This led most site staff I interviewed to the view that the results need to come from Sites, who have access to this information, rather than trial Sponsors or other organisations who may not be aware of this.

*"I think someone in my role who has access to their clinical records and knows where they are in their treatment plan and has access to their clinical letters, the clinic letters, is always the best person to do it."* CLTCI04: Trial coordinator, large site

## How close are relationships between site staff and participants likely to be?

Participants had been taking part ICON8 for between five to eight years, with face-to-face follow-up visits initially every 6 weeks straight after treatment, reducing to 3 monthly after 9 months, then 6 monthly after two years. During this time, some patients had developed a very close relationship with their site staff, particularly research nurses. This was especially the case at small and medium sites where the research nurse had been the same throughout the course of the patient's trial experience.

Patients' relationship with site staff seemed to affect satisfaction with how results were communicated; some questionnaire respondents explicitly cited it as a reason for their satisfaction. Nearly all the patients I interviewed who said they had a close relationship with their site staff were satisfied with how the results were shared.

*"My oncologist and research nurse have been excellent and I respect their dissemination of information, as it is on a personal level"* FSQI02: Patient, small site

Some patients with a close relationship with site staff would have preferred to receive directly from them, rather than via a printed summary or webpage, but were still satisfied with the way the received results, and recognised that this personal approach may not be the best for patients without that close relationship.

*"I think they should do them face to face really. I don’t know if that’s… I mean, the thing is, you build up a relationship with your trial nurses because we see them quite regularly or every time we go for a hospital appointment. So, I think it would be really nice if they presented that themselves, obviously backed up with information. I think because you’re feeling vulnerable anyway and I think if you’ve already built up a relationship with people, then it’s easier to talk to them."* DMI01: patient, medium site, close relationship with site staff

*"I don’t know, because we are a smaller centre and our numbers don’t tend to be like a big teaching hospital, we have that more personal approach, so we know our patients very well, we know the families very well, so it makes it easier for us in that respect. I’m not saying, if it was a teaching hospital you could follow the same principles, but here, we generally have that closeness."* CSRNI01: Research nurse, small site

Patients interviewed who did not have a close relationship with site staff were less satisfied with how the results were shared. For some patients this was because they struggled to understand the results, or access them, and not having a close relationship with staff meant they did not want to contact their research nurse for clarification or to ask for the information by post. In one case the dissatisfaction was around the results summary not being detailed enough, and being perceived to be received long after the results were known.

## How many participants do sites have?

The number of trial participants at a site seemed to influence site staff's views on how results should be shared. This is linked to feasibility issues, with posting information or talking to participants being less practical if they have large number of participants, but also related to how well staff knew participants. Some staff at large sites, in particular those who had worked on ICON8 for less time, felt uncomfortable contacting participants (e.g. by telephone) as they did not know them, were not sure what their current situation was, or whether they would want to be contacted. The two site staff members who felt this would have preferred an opt-in approach, as they did not know whether their participants would want to know the results, and the work involved was time-consuming. They were also worried about reminding participants that they had cancer.

*"I guess this is quite easy for me to say though because we don’t have an awful lot of patients on the ICON8 trial, so this is not labour intensive for me and [research nurse]. It will just require me to initially send out some things, a bit like what we’ve done already basically, and she’ll follow it up with a follow-up call. It’s not really that much of a problem. If we had, like, 50 or 60 patients on this trial, that could be an issue, couldn’t it?"* GMTCI02: Trial coordinator, medium site

# Expertise and resources – what expertise and resources do you have access to for sharing results?

Our previous publication contains details of the resources required for sharing results in Show RESPECT, and site staff views on the feasibility of these approaches[2].

Patients in Show RESPECT were conscious of the resource limitations of the National Health Service, and, for some, this affected their views on how they would prefer the results to be shared, balancing their own desire for personalised information with a desire not to waste resources.

*"I wouldn't have minded face-to-face, I suppose if you’ve got questions that’s useful but it’s a lot of waste of NHS money and time, when they can just send you something that you can read yourself."* BMI01: Patient, medium site

# Communication tools – which ones will you use?

Show RESPECT found that a posted printed summary in addition to a link to a webpage improved participant satisfaction compared to the webpage link without the printed summary. Information on patient and site staff views on the communication tools used in Show RESPECT, including their contents, language, visual appearance and giving participants a choice of tools have been described previously[3].

# Timing - when should results be shared with participants?

There is often a substantial gap between when trial teams first know the results (internally), when they are first presented at a scientific conference, and when they are published in a peer reviewed journal. Media coverage may take place around presentation or publication of the results, or not at all. The question of when, during this often lengthy process, the results should be shared with participants is difficult. Some site staff felt it should be done as soon as the team are sure of what the results were. Some were keen that participants were informed prior to the results being reported by the media, as finding out the results via the media would not be good. Where the media may cover results at the time of presentation, then the results may need to be communicated to participants first or concurrently.

*"If they were to find out that way via press coverage because you haven’t let them know that it’s going to be coming out in the public domain, then that might annoy certain individuals. You probably wouldn’t want to find out, like, put ITV on, the news has come out, and then all of a sudden the paparazzi are there talking about this trial. You’d probably be sitting there thinking, I could’ve done with this information earlier, couldn’t I?... Out of respect for the patient really, you should be telling them as early as possible, I can imagine."* GMTCI02: Trial Coordinator, medium site

Waiting for publication of the results, which may often be months or even a year, was seen by some as being too long, particularly for a condition like ovarian cancer when some patients may not be alive at the later point to find out the results.

*"As long as you are confident that is what is going to be written in the journal. You are sending this information to people who participated in the study. I think it’s better to let them know as soon as you are confident about whatever has been outlined. Like, waiting for a year, they might be dead."* BMRNI04: Research Nurse, medium site

*"I suppose my thought is that it probably would be okay to share that after the presentation, rather than waiting for it to go to a journal and go through the peer review process. Because that in itself will often add another twelve months or even longer to the timeline and that’s taking you even further out from the time the patients were actually participating in trial. I think doing that once the results are presented, and you’ve had some initial feedback from the discussions that happen at the conference would be the right time to do that. So, in a sense, it’s a question of preparing that information in parallel with the research presentation, isn’t it? Being in a position shortly after the conference to be able to take forward the dissemination to study participants."* HLCLI02: Oncologist, large site

# References

1. Annabelle South. Showing RESPECT: a mixed methods study into communicating the results of a Phase III clinical trial to trial participants. London: UCL; 2023.

2. South A, Bailey J, Bierer BE, Burnett E, Cragg WJ, Diaz-Montana C, et al. Site staff perspectives on communicating trial results to participants: Cost and feasibility results from the Show RESPECT cluster randomised, factorial, mixed-methods trial. Clinical Trials. 2023:17407745231186088. doi: 10.1177/17407745231186088.

3. South A, Joharatnam-Hogan N, Purvis C, James EC, Diaz-Montana C, Cragg WJ, et al. Testing approaches to sharing trial results with participants: The Show RESPECT cluster randomised, factorial, mixed methods trial. PLoS Medicine. 2021;18(10). doi: 10.1371/journal.pmed.1003798.
