# Supplementary material for: The SHOW RESPECT adaptable framework of considerations for planning how to share trial results with participants, based on qualitative findings from trial participants and site staff
Source: Trials. 2024 Jul 10;25:467. doi: 10.1186/s13063-024-08291-7 (PMC11234608; doi:10.1186/s13063-024-08291-7)
Supplement: Supplementary file 6 — Additional file 6: Ethics approval letter. Letters from ethics committee confirming approval for the study. [file 13063_2024_8291_MOESM6_ESM.pdf]

Amended on 11/09/2018 to address missing document in document list.

London - Chelsea Research Ethics Committee
Research Ethics Committee (REC) Bristol Centre
Level 3, Block B
Whitefriars
Lewins Mead
Bristol
BS1 2NT

Telephone: 0207 1048055
:

Please note: This is an acknowledgement letter from the REC only and does not allow you to start your study at NHS sites in England until you receive HRA Approval

20 August 2018

Mrs Annabelle South
90 High Holborn
2nd Floor
London
WC1V 6LJ

Dear Mrs South

**Study title:**	**Show RESults to Participants Engaged in Clinical Trials**
**REC reference:**	**18/LO/1011**
**Protocol number:**	**N/A**
**IRAS project ID:**	**245031**

Thank you for your letter of 17 August 2018. I can confirm the REC has received the documents listed below and that these comply with the approval conditions detailed in our letter dated 13 August 2018

Documents received

The documents received were as follows:

Amended on 11/09/2018 to address missing document in document list.

Document	*Version*	*Date*
IRAS Checklist XML [Checklist_17082018]		17 August 2018
Non-validated questionnaire [Quantitative Questionnaire - A]	v3.0	14 August 2018
Non-validated questionnaire [Quantitative Questionnaire - E]	v3.0	14 August 2018
Other [Quantitative Questionnaire - A - Tracked changes]	v3.0	14 August 2018
Other [Quantitative Questionnaire - E - Tracked changes]	v3.0	18 August 2018

Approved documents

The final list of approved documentation for the study is therefore as follows:

Document	*Version*	*Date*
Covering letter on headed paper [Cover Letter]		03 August 2018
Evidence of Sponsor insurance or indemnity (non NHS Sponsors only) [UCL Insurance]		24 July 2017
Interview schedules or topic guides for participants [Topic Guide]	v1.0	11 May 2018
IRAS Application Form [IRAS_Form_15052018]		15 May 2018
IRAS Application Form XML file [IRAS_Form_15052018]		15 May 2018
IRAS Checklist XML [Checklist_03082018]		03 August 2018
Letter from funder [Email confirming funding]		02 May 2018
Letter from sponsor [Sponsor Statement]		16 April 2018
Non-validated questionnaire [Quantitative Questionnaire - A]	v2.0	01 August 2018
Non-validated questionnaire [Quantitative Questionnaire - B]	v2.0	01 August 2018
Non-validated questionnaire [Quantitative Questionnaire - C]	v2.0	01 August 2018
Non-validated questionnaire [Quantitative Questionnaire - D]	v2.0	01 August 2018
Non-validated questionnaire [Quantitative Questionnaire - E]	v2.0	01 August 2018
Non-validated questionnaire [Quantitative Questionnaire - F]	v2.0	01 August 2018
Non-validated questionnaire [Quantitative Questionnaire - G]	v2.0	01 August 2018
Non-validated questionnaire [Quantitative Questionnaire - H]	v2.0	01 August 2018
Non-validated questionnaire [Site Quantitative Questionnaire - Form2A]	v1.0	01 August 2018
Non-validated questionnaire [Site Quantitative Questionnaire - Form2B]	v1.0	01 August 2018
Non-validated questionnaire [Site Quantitative Questionnaire - Form3]	v1.0	01 August 2018
Other [ICON8 Lay Summary of Results]	v1.0	11 May 2018
Other [Patient Update Information Sheet - A]	v1.0	11 May 2018
Other [Patient Update Information Sheet - B]	v1.0	11 May 2018
Other [Patient Update Information Sheet - C]	v1.0	11 May 2018
Other [Patient Update Information Sheet - D]	v1.0	11 May 2018
Other [Patient Update Information Sheet - E]	v1.0	11 May 2018
Other [Patient Update Information Sheet - F]	v1.0	11 May 2018
Other [Patient Update Information Sheet - G]	v1.0	11 May 2018
Other [Patient Update Information Sheet - H]	v1.0	11 May 2018

Amended on 11/09/2018 to address missing document in document list.

Other [Show RESPECT Protocol v2.0 24Jul2018 tracked changes]	v2.0	24 July 2018
Other [10 Show RESPECT PIS Qualitative Study v2.0 24Jul2018 tracked]	v2.0	24 July 2018
Other [Qualitative patient ICF (tracked)]	v2.0	24 July 2018
Other [Qualitative Contact Details Form]	v2.0	24 July 2018
Other [Qualitative Contact Details Form (tracked)]	v2.0	24 July 2018
Participant consent form [Qualitative Patient Study ICF]	v2.0	24 July 2018
Participant consent form [Show RESPECT Site Staff ICF Qualitative Study v2.0 24Jul2018 clean]	v2.0	24 July 2018
Participant information sheet (PIS) [Qualitative Study PIS]	v2.0	24 July 2018
Participant information sheet (PIS) [Site Staff Qualitative Information Sheet]	v2.0	24 July 2018
Referee's report or other scientific critique report [Funding Application Response]		13 November 2017
Research protocol or project proposal [Show RESPECT Protocol]	v2.0	24 July 2018
Summary CV for Chief Investigator (CI) [CV for CI]		11 May 2018
Summary CV for student [CV for Student]		11 May 2018
Summary CV for supervisor (student research) [CV for Supervisor]		11 May 2018
Summary, synopsis or diagram (flowchart) of protocol in non technical language [Trial summary and flowchart]	v1.0	11 May 2018
**Other [ICON8 Participant Summary]**	**v1.0**	**11 May 2018**

You should ensure that the sponsor has a copy of the final documentation for the study. It is the sponsor's responsibility to ensure that the documentation is made available to R&D offices at all participating sites.

**18/LO/1011**	**Please quote this number on all correspondence**

Yours sincerely

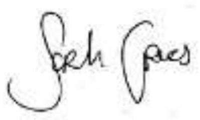

Sarah Graves
REC Manager

E-mail: nrescommittee.london-chelsea@nhs.net

Copy to: *Mrs Annabelle South*
Mrs Angela Ball, The Christie NHS Foundation Trust

:

London - Chelsea Research Ethics Committee
Research Ethics Committee (REC) Bristol Centre
Level 3, Block B
Whitefriars
Lewins Mead
Bristol
BS1 2NT

Telephone: 0207 1048055

<u>Please note</u>: This is the favourable opinion of the REC only and does not allow you to start your study at NHS sites in England until you receive HRA Approval

13 August 2018

Mrs Annabelle South
90 High Holborn
2nd Floor
London
WC1V 6LJ

Dear Mrs South

**Study title:**	**Show RESults to Participants Engaged in Clinical Trials**
**REC reference:**	**18/LO/1011**
**Protocol number:**	**N/A**
**IRAS project ID:**	**245031**

Thank you for your letter of 3 August 2018, responding to the Committee's request for further information on the above research and submitting revised documentation.

The further information has been considered on behalf of the Committee by the Chair together with Ms Watkinson.

We plan to publish your research summary wording for the above study on the HRA website, together with your contact details. Publication will be no earlier than three months from the date of this opinion letter. Should you wish to provide a substitute contact point, require further

information, or wish to make a request to postpone publication, please contact hra.studyregistration@nhs.net outlining the reasons for your request.

Confirmation of ethical opinion

On behalf of the Committee, I am pleased to confirm a favourable ethical opinion for the above research on the basis described in the application form, protocol and supporting documentation [as revised], subject to the conditions specified below.

Conditions of the favourable opinion

The REC favourable opinion is subject to the following conditions being met prior to the start of the study.

1. The Committee identified an issue with question 6 in each of the quantitative questionnaires (A-H). Eg for Grp A

6. Have you heard about the results of the ICON8 trial? Yes ☐ No ☐

The table below shows the different ways we told you about the ICON8 trial results. We would like to know how you felt about these.

Ways you may have heard about the ICON8 results:	**Webpage**
**7. Which of these do you remember being offered or given?** (Tick all that apply)	☐
**8. Which of these did you use?** (Tick all that apply)	☐
**9. Which did you prefer?** (Tick all that apply)	☐

The Committee recommend the wording is changed as only one feedback method is given and the reader may think they have incomplete information on reading the present wording that refers to multiple ways of receiving the results. The Committee recommend changing “different ways” to “way” as a fix.

You should notify the REC once all conditions have been met (except for site approvals from host organisations) and provide copies of any revised documentation with updated version numbers. Revised documents should be submitted to the REC electronically from IRAS. The REC will acknowledge receipt and provide a final list of the approved documentation for the study, which you can make available to host organisations to facilitate their permission for the study. Failure to provide the final versions to the REC may cause delay in obtaining permissions.

Management permission must be obtained from each host organisation prior to the start of the study at the site concerned.

Management permission should be sought from all NHS organisations involved in the study in accordance with NHS research governance arrangements. Each NHS organisation must confirm through the signing of agreements and/or other documents that it has given permission for the research to proceed (except where explicitly specified otherwise).

Guidance on applying for HRA and HCRW Approval (England and Wales)/ NHS permission for research is available in the Integrated Research Application System, at www.hra.nhs.uk or at http://www.rdforum.nhs.uk.

Where a NHS organisation's role in the study is limited to identifying and referring potential participants to research sites ("participant identification centre"), guidance should be sought from the R&D office on the information it requires to give permission for this activity.

For non-NHS sites, site management permission should be obtained in accordance with the procedures of the relevant host organisation.

Sponsors are not required to notify the Committee of management permissions from host organisations

Registration of Clinical Trials

All clinical trials (defined as the first four categories on the IRAS filter page) must be registered on a publically accessible database within 6 weeks of recruitment of the first participant (for medical device studies, within the timeline determined by the current registration and publication trees).

There is no requirement to separately notify the REC but you should do so at the earliest opportunity e.g. when submitting an amendment. We will audit the registration details as part of the annual progress reporting process.

To ensure transparency in research, we strongly recommend that all research is registered but for non-clinical trials this is not currently mandatory.

If a sponsor wishes to request a deferral for study registration within the required timeframe, they should contact hra.studyregistration@nhs.net. The expectation is that all clinical trials will be registered, however, in exceptional circumstances non registration may be permissible with prior agreement from the HRA. Guidance on where to register is provided on the HRA website.

It is the responsibility of the sponsor to ensure that all the conditions are complied with before the start of the study or its initiation at a particular site (as applicable).

Ethical review of research sites

NHS sites

The favourable opinion applies to all NHS sites taking part in the study, subject to management permission being obtained from the NHS/HSC R&D office prior to the start of the study (see

"Conditions of the favourable opinion" below).

Non-NHS sites

Approved documents

The final list of documents reviewed and approved by the Committee is as follows:

Document	*Version*	*Date*
Covering letter on headed paper [Cover Letter]		03 August 2018
Evidence of Sponsor insurance or indemnity (non NHS Sponsors only) [UCL Insurance]		24 July 2017
Interview schedules or topic guides for participants [Topic Guide]	v1.0	11 May 2018
IRAS Application Form [IRAS_Form_15052018]		15 May 2018
IRAS Application Form XML file [IRAS_Form_15052018]		15 May 2018
IRAS Checklist XML [Checklist_03082018]		03 August 2018
Letter from funder [Email confirming funding]		02 May 2018
Letter from sponsor [Sponsor Statement]		16 April 2018
Non-validated questionnaire [Quantitative Questionnaire - A]	v2.0	01 August 2018
Non-validated questionnaire [Quantitative Questionnaire - B]	v2.0	01 August 2018
Non-validated questionnaire [Quantitative Questionnaire - C]	v2.0	01 August 2018
Non-validated questionnaire [Quantitative Questionnaire - D]	v2.0	01 August 2018
Non-validated questionnaire [Quantitative Questionnaire - E]	v2.0	01 August 2018
Non-validated questionnaire [Quantitative Questionnaire - F]	v2.0	01 August 2018
Non-validated questionnaire [Quantitative Questionnaire - G]	v2.0	01 August 2018
Non-validated questionnaire [Quantitative Questionnaire - H]	v2.0	01 August 2018
Non-validated questionnaire [Site Quantitative Questionnaire - Form2A]	v1.0	01 August 2018
Non-validated questionnaire [Site Quantitative Questionnaire - Form2B]	v1.0	01 August 2018
Non-validated questionnaire [Site Quantitative Questionnaire - Form3]	v1.0	01 August 2018
Other [ICON8 Lay Summary of Results]	v1.0	11 May 2018
Other [Patient Update Information Sheet - A]	v1.0	11 May 2018
Other [Patient Update Information Sheet - B]	v1.0	11 May 2018
Other [Patient Update Information Sheet - C]	v1.0	11 May 2018
Other [Patient Update Information Sheet - D]	v1.0	11 May 2018
Other [Patient Update Information Sheet - E]	v1.0	11 May 2018
Other [Patient Update Information Sheet - F]	v1.0	11 May 2018
Other [Patient Update Information Sheet - G]	v1.0	11 May 2018
Other [Patient Update Information Sheet - H]	v1.0	11 May 2018
Other [Show RESPECT Protocol v2.0 24Jul2018 tracked changes]	v2.0	24 July 2018
Other [10 Show RESPECT PIS Qualitative Study v2.0 24Jul2018 tracked]	v2.0	24 July 2018
Other [Qualitative patient ICF (tracked)]	v2.0	24 July 2018
Other [Qualitative Contact Details Form]	v2.0	24 July 2018

Other [Qualitative Contact Details Form (tracked)]	v2.0	24 July 2018
Participant consent form [Qualitative Patient Study ICF]	v2.0	24 July 2018
Participant consent form [Show RESPECT Site Staff ICF Qualitative Study v2.0 24Jul2018 clean]	v2.0	24 July 2018
Participant information sheet (PIS) [Qualitative Study PIS]	v2.0	24 July 2018
Participant information sheet (PIS) [Site Staff Qualitative Information Sheet]	v2.0	24 July 2018
Referee's report or other scientific critique report [Funding Application Response]		13 November 2017
Research protocol or project proposal [Show RESPECT Protocol]	v2.0	24 July 2018
Summary CV for Chief Investigator (CI) [CV for CI]		11 May 2018
Summary CV for student [CV for Student]		11 May 2018
Summary CV for supervisor (student research) [CV for Supervisor]		11 May 2018
Summary, synopsis or diagram (flowchart) of protocol in non technical language [Trial summary and flowchart]	v1.0	11 May 2018

Statement of compliance

The Committee is constituted in accordance with the Governance Arrangements for Research Ethics Committees and complies fully with the Standard Operating Procedures for Research Ethics Committees in the UK.

After ethical review

<u>Reporting requirements</u>

The attached document *"After ethical review – guidance for researchers"* gives detailed guidance on reporting requirements for studies with a favourable opinion, including:

- Notifying substantial amendments
- Adding new sites and investigators
- Notification of serious breaches of the protocol
- Progress and safety reports
- Notifying the end of the study

The HRA website also provides guidance on these topics, which is updated in the light of changes in reporting requirements or procedures.

User Feedback

The Health Research Authority is continually striving to provide a high quality service to all applicants and sponsors. You are invited to give your view of the service you have received and the application procedure. If you wish to make your views known please use the feedback form available on the HRA website:
http://www.hra.nhs.uk/about-the-hra/governance/quality-assurance/

HRA Training

We are pleased to welcome researchers and R&D staff at our training days – see details at http://www.hra.nhs.uk/hra-training/

**18/LO/1011**	**Please quote this number on all correspondence**

With the Committee's best wishes for the success of this project.

Yours sincerely

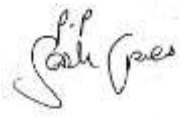

Dr Michael Schachter
Chair

Email:nrescommittee.london-chelsea@nhs.net

Enclosures: “After ethical review – guidance for researchers” *[SL-AR2]*

Copy to: *Miss Cara Purvis*
Mrs Angela Ball , The Christie NHS Foundation Trust
